# Short chain fatty acids induced the type 1 and type 2 fimbrillin-dependent and fimbrillin-independent initial attachment and colonization of *Actinomyces oris* monoculture but not coculture with streptococci

**DOI:** 10.1186/s12866-020-01976-4

**Published:** 2020-10-31

**Authors:** Itaru Suzuki, Takehiko Shimizu, Hidenobu Senpuku

**Affiliations:** 1 Department of Bacteriology I, National Institute of Infectious Diseases, 1-23-1 Toyama, Shinjuku-ku, Tokyo, 162-8640 Japan; 2 Department of Pediatric Dentistry, Nihon University Graduate School of Dentistry at Matsudo, Chiba, Japan

**Keywords:** Biofilm, Initial colonization, *Actinomyces oris*, Fimbrillin, SCFAs

## Abstract

**Background:**

*Actinomyces oris* is an early colonizer and has two types of fimbriae on its cell surface, type 1 fimbriae (FimP and FimQ) and type 2 fimbriae (FimA and FimB), which contribute to the attachment and coaggregation with other bacteria and the formation of biofilm on the tooth surface, respectively. Short-chain fatty acids (SCFAs) are metabolic products of oral bacteria including *A. oris* and regulate pH in dental plaques. To clarify the relationship between SCFAs and fimbrillins, effects of SCFAs on the initial attachment and colonization (INAC) assay using *A. oris* wild type and fimbriae mutants was investigated. INAC assays using *A. oris* MG1 strain cells were performed with SCFAs (acetic, butyric, propionic, valeric and lactic acids) or a mixture of them on human saliva-coated 6-well plates incubated in TSB with 0.25% sucrose for 1 h. The INAC was assessed by staining live and dead cells that were visualized with a confocal microscope.

**Results:**

Among the SCFAs, acetic, butyric and propionic acids and a mixture of acetic, butyric and propionic acids induced the type 1 and type 2 fimbriae-dependent and independent INAC by live *A. oris*, but these cells did not interact with streptococci. The main effects might be dependent on the levels of the non-ionized acid forms of the SCFAs in acidic stress conditions. GroEL was also found to be a contributor to the FimA-independent INAC by live *A. oris* cells stimulated with non-ionized acid.

**Conclusion:**

SCFAs affect the INAC-associated activities of the *A. oris* fimbrillins and non-fimbrillins during ionized and non-ionized acid formations in the form of co-culturing with other bacteria in the dental plaque but not impact the interaction of *A. oris* with streptococci.

## Background

Initial colonizers such as the oral bacteria *Actinomyces* spp*., Streptococcus* spp*., Veillonella* spp. and *Neisseria* spp. play important roles in biofilm formation in human oral cavities through their interactions with other bacteria on the tooth surface [1–4]. Biofilm communities on the tooth surface are polymicrobial [5, 6], and more than 60 to 90% of the biofilm bacteria in salivary components that coat the enamel surface are streptococci [7, 8]. *Actinomyces* spp. aggregate with *Streptococcus* spp. during the progression of dental caries and contribute to periodontal diseases [9–11]. *Actinomyces oris*, which was formerly referred to as *Actinomyces naeslundii* genospecies 2 [7, 12], is considered an initial colonizer that interacts with other bacteria in the oral cavity [13]. *A. oris* induces the coaggregation of the early colonizers *Streptococcus gordonii* and *Streptococcus sanguinis* with the intermediate colonizer *Fusobacterium nucleatum* in oral biofilms [1].

Human salivary components, such as statherin [14], proline-rich proteins (PRPs) [15, 16], gp-340 [17, 18] and mucin (MUC7) [19, 20], control the attachment and colonization of oral bacteria on the tooth surface [21]. *A. naeslundii,* which was formerly referred to as genospecies 1*,* and *A. oris* bind to PRPs and statherin, a phosphate-containing protein in salivary components [1]. *A. oris* have two functionally and antigenically distinct types of important fimbriae on the cell surface: type 1 and type 2 fimbriae are formed by the shaft fimbrillins, FimP and FimA, and the tip fimbrillins, FimQ and FimB, respectively [22]. Type 1 fimbriae mediate the attachment to PRPs on the tooth surface [23], and type 2 fimbriae mediate the coaggregation and late biofilm formation with streptococci [24–27].

Short-chain fatty acids (SCFAs) are secreted by oral bacteria such as *Porphyromonas gingivalis*, *F. nucleatum* [28, 29] and *Veillonella parvula* [30]. In healthy human volunteers, the concentrations of acetic, lactic, propionic, formic, butyric and valeric acids among the total SCFAs detected in saliva were found to be 6.0 ± 3.5 mM, 1.2 ± 1.9 mM, 1.0 ± 0.8 mM, 0.5 ± 0.5 mM, 0.3 ± 0.4 mM and 0.05 ± 0.2 mM, respectively [31]. In another report, the maximum concentrations of butyric (8.8 mM), propionic (33.7 mM), acetic (52.6 mM) and formic acids (5.8 mM) were also detected in dental plaques from caries-free and caries-susceptible young subjects [32]. These starved plaque fluid samples predominantly contained butyric, propionic and acetic acids with high pKa values (dissociation constant) of 4.82, 4.87 and 4.76, respectively, and the total concentration of the mixture of all acids was 95.1 mM (94.3%). These acids effectively buffer the pH in the range considered in the study (i.e., pH 6 to 4). In the Stephan curve [33], acid production is just one of many biological processes that occur within human plaques exposed to sugar. The Stephan curve has played a dominant role in caries research over the past several decades. In oral biofilm bacteria, organic acids are mainly generated by the fermentation of sugars during or after the intake of food, including sugar, and reduce the pH to levels lower than 5.0 [34, 35]. However, fresh saliva is produced to neutralize the reduced pH of the biofilm. Constant stimulation by a low pH may affect biofilm bacteria on the tooth surface.

As we have previously reported, the rate of biofilm formation by *A. naeslundii* X600 cells was upregulated by 6.25 mM butyric acid, 3.13 mM propionic acid and 3.13 mM valeric acid compared to the rate in the control (no SCFA) in human saliva-coated 96-well microtiter plates [36]. When biofilm cells were subjected to SDS-PAGE and western blot analysis, the results demonstrated that this upregulation was mediated by the heat shock protein GroEL [36]. Another report showed that the number of biofilms consisting of *A. naeslundii* X600 cells generated from initial attachment cells in a flow cell system was also increased by treatment with 60 mM butyric acid [37]. These reports suggested that the effects of SCFAs, including butyric acid, might induce the potentiation of the cell status required for initial cell attachment, colonization and biofilm formation [37, 38]. A total SCFA concentration of 60 mM and a low pH (pH 4.7) were required for the initial attachment of and biofilm formation by *A. naeslundii* [37]. SCFAs generated after the fermentation of sugars in the biofilm may affect the recolonization and spreading of *Actinomyces* spp. However, the relationship between SCFAs and the fimbrillin-dependent initial attachment of *A. oris*, which belongs to a different type than *A. naeslundii*, is not clear. *A. oris* shows fimbrillin-dependent activities related to cell adherence and aggregation in cocultures of *A. oris* and streptococci. The effect of SCFAs on cocultures of *A. oris* and oral streptococci and the relationship between fimbrillin and GroEL are also interesting issues.

In this study, we performed initial attachment and colonization (INAC) assays to determine the relationship between fimbriae and SCFAs in *A. oris*. We clearly determined that a total SCFA concentration of 60 mM involving acids with high pKa values (butyric, propionic and acetic acids) promoted type 1 (FimP and FimQ) and type 2 (FimA and FimB) fimbrillin-dependent INAC by live *A. oris* cells. However, the promotion of the FimA-dependent INAC of *A. oris* by SCFAs did not mediate the interactions of *A. oris* with *S. sanguinis*. FimA-independent INAC was associated with GroEL under the stress conditions induced by 60 mM butyric acid treatment. These results provide new evidence regarding the mechanisms of the fimbrillin-dependent and fimbrillin-independent INAC of *A. oris* on a human saliva-coated surface and the communication of *A. oris* with the oral bacteria that produce SCFAs.

## Results

### Effect of SCFAs on the INAC of *A. oris*

**Fig. 1:**
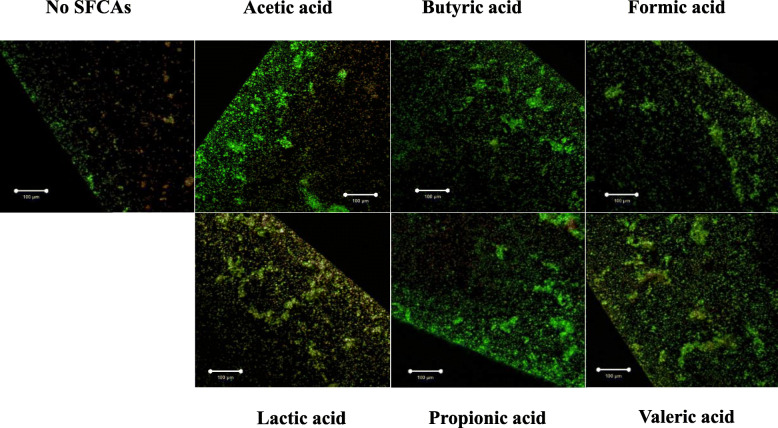
Effect of SCFAs on the initial attachment and colonization of *A. oris. A. oris* MG1 was cultivated in TSB supplemented with or without 60 mM SCFAs (acetic, butyric, formic, lactic, propionic and valeric acids) for 1 h. The attached and colonized cells were observed at the edges of the wells. INAC was observed by confocal microscopy. Merged images of the live cells (green colour) and dead cells (red colour) are presented in the pictures, in which the effects of butyric acid on INAC were observed. Scale bars indicate 100 μm. All CLSM images were obtained with a 10× objective. Representative data from more than three independent experiments are presented in each panel

**Fig. 2:**
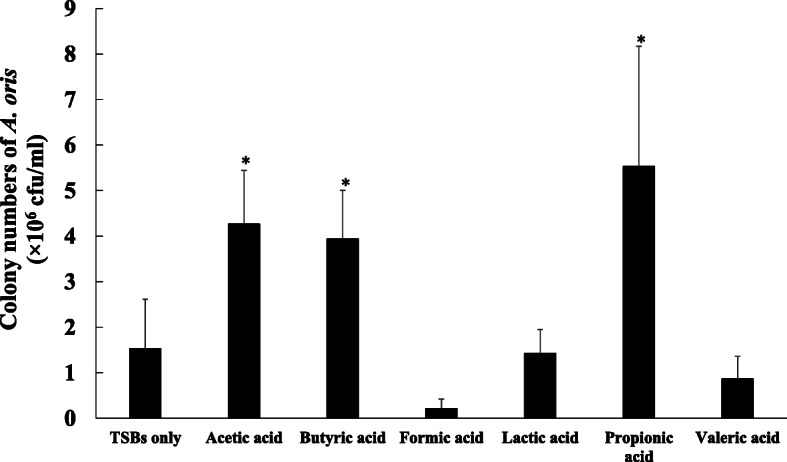
Number of live *A. oris* cells that were initially attached and colonized after treatment with SCFAs. *A. oris* MG1 was cultivated in TSB supplemented with or without 60 mM SCFAs (acetic, butyric, formic, lactic, propionic and valeric acids) for 1 h. The attached and colonized cells were harvested with a scraper, were pipetted, and placed onto BHI agar plates. These cells were counted on BHI agar plates after 48 h. The data represent the mean ± standard deviation (SD) of triplicate experiments. The experiments were performed three times, with similar results obtained in each replicate. The asterisks indicate a significant difference between the two groups (Student’s *t*-test, *p*-value < 0.05, no SCFAs vs. SCFAs)

To confirm the effects of butyric acid on the INAC of *A. oris*, various concentrations of butyric acid were applied, and the dose-dependent effects of the butyric acid on the INAC were visually observed by confocal microscopy (S1 Fig). INAC was stimulated by levels of butyric acid higher than 30 mM (S1 Fig). Compared to the other concentrations, 60 mM butyric acid showed the greatest INAC. Other SCFAs treated at 60 mM concentrations also increased the initial attachment and aggregates of *A. oris* compared to the control (no SCFA) (Fig. 1). To confirm that the number of bacterial live cells was reflected in the amount of stimulation, the attached cells were removed and added onto BHI agar plates. After the cells were incubated for 48 h, the number of colonies was counted. Treatment with acetic acid, butyric acid or propionic acid significantly increased the number of cells compared to that observed in the no-SCFA control (*p*-value < 0.05), while the number of cells in the lactic acid and valeric acid treatment groups did not differ from that of the control condition (Fig. 2). Compared to the control, formic acid treatment led to a decreased number of cells. Therefore, the biological activities of acetic, butyric and propionic acids were different from those of formic, lactic and valeric acids.

**Fig. 3:**
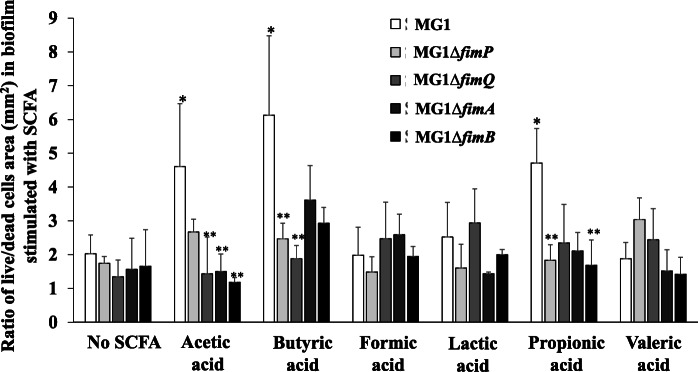
Effect of SCFAs on the INAC of the wild-type *A. oris* and the fimbrillin mutants. The INAC of the wild-type *A. oris* MG1 and the Δ*fimP*, Δ*fimQ*, Δ*fimA* and Δ*fimB* mutants were observed under conditions with or without SCFAs. The attached and colonized cells were stained using a LIVE/DEAD staining kit and observed by CLSM at the edges of the wells. All CLSM images were obtained with a 10× objective. TSB without SCFAs, TSB with 60 mM acetic acid, TSB with 60 mM butyric acid, TSB with 60 mM formic acid, TSB with 60 mM lactic acid, TSBs with 60 mM propionic acid and TSB with 60 mM valeric acid were used as the media for the INAC assays for the wild-type *A. oris* MG1 and the MG1 Δ*fimA* mutant strain. Live and dead cell areas were analysed by ImageJ from CLSM images, and the ratios of the live areas to the dead cell areas were calculated by ImageJ. The data represent the mean ± standard deviation (SD) of three independent experiments. The asterisks indicate a significant difference between the two groups (Student’s *t*-test, *p*-value < 0.05, *: no SCFAs vs. SCFAs, **: MG1 vs. each MG1 mutant)

To explore the roles of fimbrillin in the initial attachment of *A. oris,* MG1 fimbrillin mutants (Δ*fimP,* Δ*fimQ,* Δ*fimA* and Δ*fimB*) were used in an INAC assay of cells treated with 60 mM SCFAs. The INAC of the fimbrillin-mutant *A. oris* was visibly increased by stimulation of SCFAs compared with no stimulation, but reduced compared to those of the wild-type MG1 control, which were treated with SCFAs (S2 Fig). In particular, the Δ*fimA* cells showed poor attachment and colonization. To clear whether live and dead cells are dominantly adhered on the saliva-coated plate surface, the percentages of the areas with live (green colour) and dead cells (red colour) in all the pictures obtained by the CLSM were calculated using ImageJ. The ratios of the live cell areas to the dead cell areas were re-calculated. The ratios were significantly higher in the acetic-, butyric- and propionic acid-treated MG1 cells than in the untreated MG1 cells (*p*-value < 0.05) (Fig. 3). However, the ratios of the live cell areas to the dead cell areas induced by the formic, lactic and valeric acid treatments and the controls were equivalent. The increased initial attachment of *A. oris* was visually observed in all SCFA-treated wild-type cells (Fig. 1); however, significant increases in the number of attached live cells and the predominance of the live cell areas to the dead cell areas were observed with the acetic, butyric and propionic acid treatments but not with formic, lactic and valeric acid treatments (Figs. 1 and 3). Therefore, the cells that were observed to be initially attached may include more adherence and aggregation of dead cells after formic, lactic or valeric acids treatments than after acetic, butyric or propionic acids treatments. In the fimbrillin mutants, the ratios of the live cells areas to the dead cells were totally reduced as compared with wild-type in acetic, butyric and propionic acids (Fig. 3). With acetic and butyric acid stimulation, the ratios of the live cell areas to the dead cell areas were significantly lower in the Δ*fimP*, and Δ*fimP* and Δ*fimQ* than those in the wild-type strain, respectively (*p*-value < 0.05) (Fig. 3). While the ratios were significantly lower in the Δ*fimA* and Δ*fimB* than the wild-type in acetic acid, the differences between the wild-type and mutant strains were not significant after butyric acid treatment. Therefore, type 1 and type 2 fimbrillins were both required for the acetic acid-induced increase in INAC, but type 1 fimbrillin was mainly necessary for the spread of the live cell attachment area in butyric acid. With propionic acid, the ratios of the live cell areas to the dead cell areas were significantly lower in the Δ*fimP* and Δ*fimB* than in the wild-type strain (*p*-value < 0.05) (Fig. 3). The ratios were also lower in Δ*fimQ* and Δ*fimA*; however, the differences between the wild-type and mutant strains were not significant.

### Effects of low pH and butyric acid on INAC

**Fig. 4:**
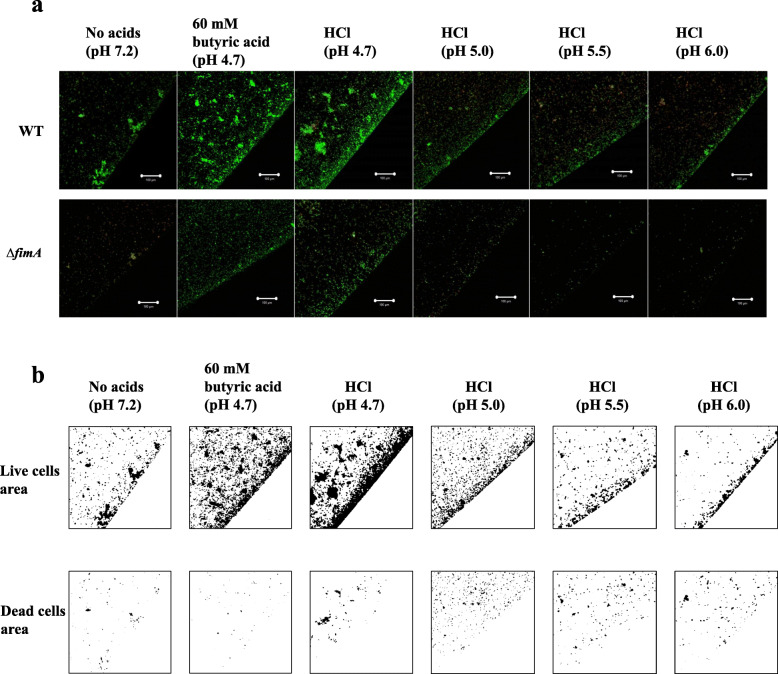
Effects of 60 mM butyric acid and HCl-mediated pH conditions on INAC. **a** INAC of the wild-type *A. oris* MG1 and the MG1 Δ*fimA* mutant was observed in conditions with or without 60 mM butyric acid and at various pH values (pH 4.7, 5.0, 5.5 and 6.0) prepared with HCl. The attached and colonized cells were stained using a LIVE/DEAD staining kit and observed by CLSM at the edges of the wells. All CLSM images were obtained with a 10× objective. Scale bars indicate 100 μm. Images were further analysed to determine the areas of live cells and dead cells in MG1 using ImageJ. **b** Representative data from more than three independent experiments are presented in each panel

**Fig. 5:**
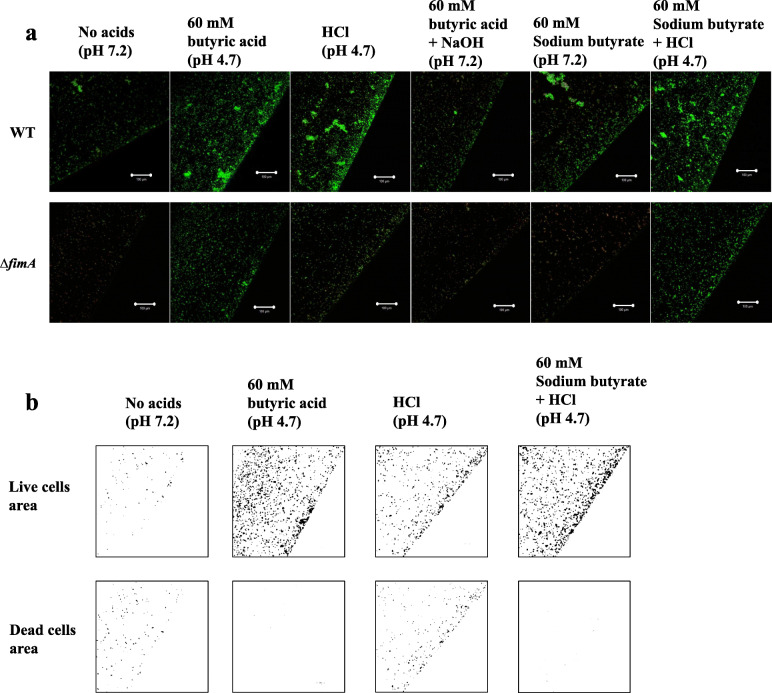
Effects of 60 mM butyric acid with lowered pH on INAC. **a** INAC of the wild-type *A. oris* MG1 and the MG1 Δ*fimA* mutant strains were observed under the control condition, 60 mM butyric acid condition, pH 4.7 condition prepared with HCl, pH 7.2 condition prepared with NaOH, 60 mM sodium butyrate condition and pH 4.7 condition with 60 mM sodium butyrate prepared with HCl. The attached and colonized cells were stained using a LIVE/DEAD staining kit and observed by CLSM at the edges of the wells. All CLSM images were obtained with a 10× objective. Scale bars indicate 100 μm. **b** Images were further analysed to determine the areas of live cells and dead cells in MGΔ*fimA* using ImageJ. Representative data from more than three independent experiments are presented in each panel

The culture of the TSB supplemented with 60 mM butyric acid had a pH of 4.7 and included both the ionized and non-ionized acid forms because 50% of butyric acid exists in the ionized form and 50% of butyric acid exists in the non-ionized form at pH conditions near pH 4.82 (the pKa for butyric acid). However, the ratio of the non-ionized form to the ionized form is reduced by increasing the pH. Treatment with 60 mM butyric acid induced an increase in the INAC of *A. oris* compared with the INAC rate induced by concentrations of butyric acid lower than 60 mM (S1 Fig). There is a possibility that an increase in the non-ionized forms affects the INAC at pH 4.7 during the 60 mM butyric acid treatment. Various pH conditions were generated with the strong acid HCl, and these changes resulted in complete conversion to only the ionized form because the pKa was − 8. The effects of these conditions were compared with those of the butyric acid treatment used in the INAC assay. The results showed that the primary pH 4.7 condition set with HCl reduced the INAC of the *A. oris* MG1 compared with that after 60 mM butyric acid treatment (Fig. 4a). However, the pH 4.7 condition, as prepared with HCl, induced greater INAC than those found with the other high pH conditions (pH 5.0, pH 5.5 and pH 6.0) and with no added acid. In the analyses of the live and dead cell areas of the MG1, the majority of cells were alive after the 60 mM butyric acid treatment under the pH 4.7 condition as prepared with HCl, but more dead cells were also observed under the conditions prepared with HCl than in the conditions created with 60 mM butyric acid or without added acid (Fig. 4b). However, the ionized acid form in the pH 4.7 condition, as prepared with HCl, was associated with positive effects on the INAC of live and dead MG1 cells. In contrast, the fimbriae-dependent INAC by live MG1 cells stimulated with 60 mM butyric acid was mainly associated with the presence of the non-ionized acid form because the pH 4.7 conditions prepared with HCl resulted in the total conversion to the ionized acid form, resulting in an increased number of dead cells. All mutants showed reduction of the INAC ratio of live and dead MG1 cells in acetic, butyric and propionic acids. To clear these mechanisms, we selected Δ*fimA* and compared with wild-type in various conditions of pH. For the MG1 Δ*fimA,* the pH 4.7 condition prepared with HCl induced greater INAC than did the other pH conditions and the no-acid condition but a lower INAC than the 60 mM butyric acid condition (Fig. 4a). To elucidate the effects of the low pH with butyric acid, a pH 7.2 condition prepared with NaOH in 60 mM butyric acid, a pH 7.2 condition in sodium butyrate, and a pH 4.7 condition prepared with HCl in sodium butyrate were evaluated with an INAC assay of the MG1 and MG1Δ*fimA*. Compared with the 60 mM butyric acid (pH 4.7) condition, the INAC was reduced by the increase in pH (pH 7.2) in the 60 mM butyric acid condition prepared by NaOH, and in contrast, the INAC was increased by the decrease in pH (pH 4.7) in the 60 mM sodium butyrate condition prepared by HCl in the MG1 and MG1Δ*fimA* (Fig. 5a). The ionized form was relatively increased in the NaOH-mediated pH conditions from 4.7 to 7.2 in 60 mM butyric acid because the ionized form is the main form present in pH conditions above the pKa. In contrast, positive effects on the INAC of the MG1 and MG1Δ*fimA* were observed when the non-ionized form was increased in the HCl-mediated pH conditions from 7.2 to 4.7 in 60 mM sodium butyrate. In the analyses of the live and dead cell areas of MG1Δ*fimA*, live cells were primarily observed in the 60 mM butyric acid and pH 4.7 condition prepared with HCl in 60 mM sodium butyrate, but more dead cells were also observed under the pH 4.7 condition prepared with HCl than those found under the other pH 4.7 conditions prepared with butyric acid and pH 4.7 condition prepared with HCl in 60 mM sodium butyrate (Fig. 5a). The results in the pH 4.7 condition prepared with HCl in sodium butyrate were similar to those in 60 mM butyric acid. Taken together, these data indicate that the fimbriae-independent INAC by live MG1Δ*fimA* cells stimulated with 60 mM butyric acid might be primarily associated with the non-ionized acid form.

### Effects of the mixture of acetic, butyric and propionic acids on INAC

**Fig. 6:**
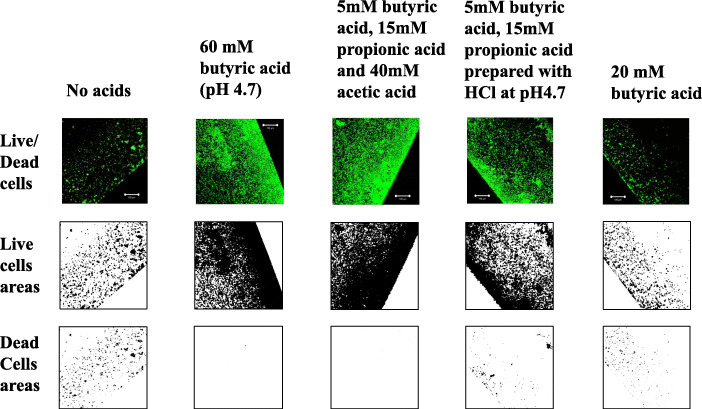
Effects of a mixture of acetic acid, propionic acid and butyric acid on INAC. INAC areas of wild-type *A. oris* MG1 and MG1 Δ*fimA* by live cells and with dead cells were observed in the control, with no acid; with 60 mM butyric acid treatment; mixture of 5 mM butyric acid, 15 mM propionic acid and 40 mM acetic acid treatment; and under the pH 4.7 condition prepared with HCl in mixture of 5 mM butyric acid and 15 mM propionic acid, and 20 mM butyric acid. The attached and colonized cells were stained using a LIVE/DEAD staining kit and were observed by CLSM at the edges of the wells. All CLSM images were obtained with a 10× objective. Images were further analysed to determine the live and dead cell areas in MG1 using ImageJ. Representative data from more than three independent experiments are presented in each panel

Common properties include the presence of 50% of the non-ionized forms in 60 mM acetic, 60 mM butyric and 60 mM propionic acids. Therefore, each non-ionized acid may have the same effects on the INAC of *A. oris*. In dental plaques, acetic, propionic and butyric acids are mixed and the mix acids may affect biofilm formation [31]. To confirm the effects of the mixture of butyric, propionic and acetic acids on INAC, 5 mM butyric acid, 15 mM propionic acid and 40 mM acetic acid, as referenced in a clinical data from a previous paper [32] and active level of concentration of each acid in this study, were used to prepare a total sample of mixed SCFAs of 60 mM and to apply in the INAC assay. The findings indicated that this mixture, as well as 60 mM butyric acid, induced extremely large INAC areas of live *A. oris* MG1 and MG1Δ*fimA* cells (Fig. 6). In contrast, the INAC areas of *A. oris* MG1 and MG1Δ*fimA* dead cells were low for both the 60 mM and the full mixture. To observe whether a total of 60 mM of high pKa acids (acetic, butyric and propionic acids) was necessary to induce the INAC areas, a pH 4.7 condition prepared by HCl (treatments with low pKa acids instead of acetic acid) in a mixture of 5 mM butyric acid and 15 mM propionic acid were assessed with the INAC assay, and the results were compared with those of the full mixture of high pKa acids. The INAC areas of the live *A. oris* MG1 and MG1Δ*fimA* cells were decreased in the pH 4.7 condition prepared by HCl in a mixture of 5 mM butyric acid and 15 mM propionic acid compared with the full mixture (Fig. 6). In contrast, the INAC areas of the *A. oris* MG1 and MG1Δ*fimA* with dead cells were increased in the pH 4.7 condition prepared by HCl in a mixture of 5 mM butyric acid and 15 mM propionic acid compared with the full mixture. These findings indicate that use of HCl instead of acetic acid led to decreased and increased INAC areas of live cells and dead cells, respectively. Twenty mM butyric acid (pH 6.1) induced slightly smaller INAC areas of *A. oris* wild-type and MG1Δ*fimA* consisting of live cells and dead cells than did the control condition of no acid. This indicated that pH was higher in 20 mM butyric acid than 60 mM (pH 4.7) and not enough for the stimulation to the induction of INAC cells.

### Effect of SCFAs on INAC and the interactions between *A. oris* and *S. sanguinis*

Type 2 fimbriae mediate coaggregation and biofilm formation with other bacteria. Based on the results above, we hypothesized that *A. oris* FimA would be an important factor in the SCFA-stimulated INAC of *A. oris* in the presence of *S. sanguinis* or *S. gordonii.* To determine whether treatment by individual SCFA at 60 mM would affect the INAC of a culture of *A. oris* MG1 or MG1 Δ*fimA* strain mixed with streptococci, we performed INAC assays using *A. oris* and streptococci. *S. salivarius* was used for the INAC assay as a control.

A significant increase in INAC was observed in *A. oris* MG1 and *S. sanguinis* but not in *A. oris* MG1 with *S. gordonii* or *S. salivarius* (S3 Fig). Co-aggregation of MG1 with *S. sanguinis* was presented in various areas. In contrast, the INAC was visibly reduced in the *A. oris* MG1 Δ*fimA* with *S. sanguinis, S. gordonii or S. salivarius* compared to the wild-type *A. oris* MG1 and other streptococci (S4 Fig). The INAC areas of *A. oris* MG1 and *S. sanguinis* were clearly reduced by the addition of the SCFAs (S5 Fig). Furthermore, the INAC area of the *A. oris* MG1 Δ*fimA* with *S. sanguinis* was slightly reduced by the addition of SCFAs (S6 Fig). This INAC was also enhanced for the culture of *A. oris* MG1 mixed with *S. sanguinis* compared to the monoculture of *S. sanguinis* (S7 Fig). Moreover, SCFAs clearly inhibited the INAC of a monoculture of *S. sanguinis* (S7 Fig). However, the INAC area of a culture of the *A. oris* MG1 Δ*fimA* mixed with *S. sanguinis* was slightly increased compared to the monocultures of the *A. oris* MG1 Δ*fimA* or *S. sanguinis* (S7 Fig). These results demonstrate that SCFAs inhibited the INAC of a culture of *A. oris* mixed with *S. sanguinis.* We considered that the INAC of *S. sanguinis* may also be inhibited by SCFAs. In contrast, the INAC of *A. oris* was enhanced by SCFAs. *A. oris* FimA interacts with *S. sanguinis* [26], but this interaction is not induced by SCFAs, according to the INAC assay in this study In addition to FimA, other factors of *A. oris* may interact with *S. sanguinis* when the cells are treated with SCFAs*.*

**Fig. 7:**
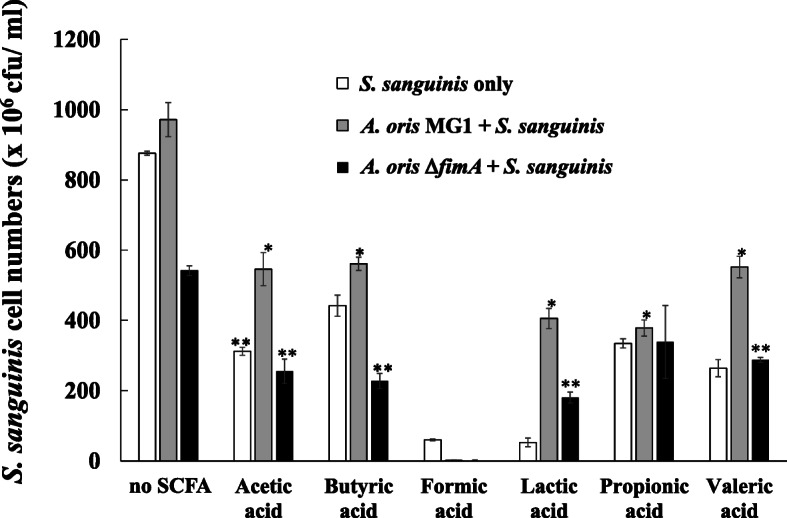
Number of *S. sanguinis* cells in *S. sanguinis* monocultures and in cultures of *S. sanguinis* mixed with *A. oris* MG1 or MG1 Δ*fimA.* The number of *S. sanguinis* colonies on mitis-salivarius agar plates was counted in a monoculture of *S. sanguinis* and in a culture of *S. sanguinis* mixed with *A. oris* MG1 or MG1 Δ*fimA* strains. The data represent the mean ± standard deviation (SD) of triplicate experiments. The experiments were performed three times, with similar results obtained in each replicate. The asterisks indicate a significant difference between the two groups (Student’s *t*-test, *p*-value < 0.05, * no SCFA vs. SCFA, ** MG1 vs. MG1.Δ*fimA*)

To determine the number of *S. sanguinis* cells in a monoculture of *S. sanguinis* and the number in a culture of *S. sanguinis* mixed with either *A. oris* MG1 or MG1Δ*fimA*, in conditions with or without SCFAs, the attached and colonizing cells were removed from the plates using a cell scraper. The cells were suspended in sterile PBS and plated onto mitis-salivarius selective agar plates. The stimulation of acetic, butyric and propionic acids increased CFU of *A. oris* MG1 (Fig. 1). However, the number of live *S. sanguinis* cells after the SCFA treatments was significantly lower than that in the control condition (*p*-value < 0.05) (Fig. 7). However, the number of live *S. sanguinis* cells in cocultures of *A. oris* MG1 and *S. sanguinis* was significantly higher than that in the *S. sanguinis* monocultures (*p*-value < 0.05) when cells were treated with acetic acid, butyric acid, lactic acid, propionic acid or valeric acid but not when the cells were treated with formic acid (Fig. 7). In contrast, the number of live *S. sanguinis* cells was significantly lower in cocultures of *A. oris* Δ*fimA* and *S. sanguinis* than in cocultures of *A. oris* MG1 and *S. sanguinis* without SCFA treatment and with butyric acid, lactic acid, or valeric acid treatments (*p*-value < 0.05) but not with formic acid or propionic acid treatments.

### Effects of anti-GroEL antibodies on SCFA-stimulated INAC

**Fig. 8:**
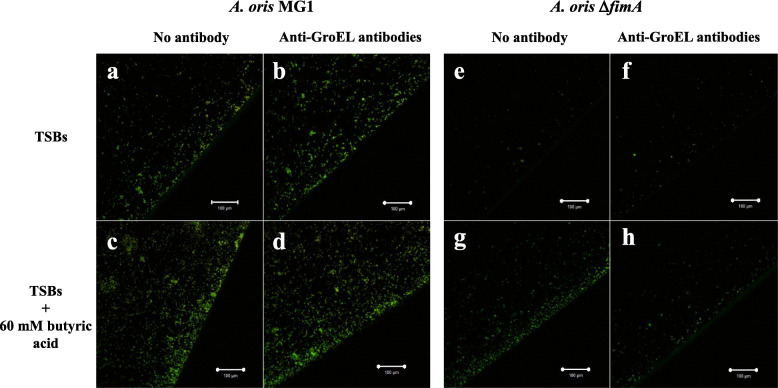
Effects of anti-GroEL antibodies on the INAC of *A. oris.* TSB only (**a**, **b**, **e** and **f**) and with 60 mM butyric acid (**c**, **d**, **g** and **h**) were applied to the cultures for the INAC assays of the wild-type *A. oris* MG1 and the MG1 Δ*fimA* strains. Anti-GroEL rabbit antibodies were diluted 1/4000 and applied to the cultures (**c**, **d**, **g** and **h**). The attached and colonized cells were observed at the edges of the wells. INAC areas were observed by confocal microscopy. Merged images of the live cells (green colour) and dead cells (red colour) are presented in the pictures, in which the effects of butyric acid on the INAC are observed. All CLSM images were obtained with a 10× objective. Scale bars indicate 100 μm. Representative data from more than three independent experiments are presented in each panel

To observe the role of GroEL, which is associated with the initial attachment of *A. naeslundii* [37], in butyric acid-induced INAC, anti-GroEL antibodies were added to the cultures that were subjected to INAC assay. This antibody could not inhibit the INAC of the *A. oris* MG1 stimulated with butyric acid (Fig. 8 left). The INAC of *A. oris* MG1 Δ*fimA* was slightly enhanced by butyric acid, and this increase was inhibited by the anti-GroEL antibody (Fig. 8 right). Moreover, this increase in INAC was not inhibited by the control antibodies (anti-GBPC polyclonal rabbit antibodies; data not shown). Therefore, GroEL plays a key role in the INAC of the *A. oris* MG1 Δ*fimA* strain stimulated with butyric acid.

## Discussion

The 60 mM butyric acid, 60 mM propionic acid and 60 mM acetic acid treatments resulted in larger numbers of live *A. oris* cells and larger areas of type 1- and type 2-dependent INAC than those involving the other SCFAs. These acids are anionic acids with high pKa values and act as effective buffers during the production of organic acids, including those with low pKa values (e.g., lactic acid) [39]. The concentrations of butyric and propionic acids have been significantly associated with clinical measures of disease severity (e.g., pocket depth, attachment level), inflammation (e.g., subgingival temperature, % of sites bleeding when probed), and the total microbial load (all *p*-values < 0.05) [28]. Butyric and propionic acids at concentrations of 6.25 mM each promote FimA-dependent biofilm formation, including an increase in dead cells in vitro, as determined by experimental flow cell systems after 48 h [38]. Acids with high pKa values, such as butyric, propionic and acetic acids, may exhibit a specific ability to stimulate the INAC of both *A. naeslundii* and *A. oris* when applied at high concentrations (i.e., 60 mM for at least 1 h). Moreover, these SCFAs may mediate the biofilm formation of *A. naeslundii* and the FimA-dependent biofilm formation of *A. oris* at low concentrations (i.e., 6.25 mM for 6 h or more under induction with various SCFAs or for 48 h in conditions without these metabolites). Therefore, the combination of a high concentration of SCFAs with a short incubation time or a low concentration of SCFAs with a long incubation time might be the key to stimulating the INAC of *Actinomyces* spp. Additionally, SCFAs at 60 mM show a pH of 4.7; however, at 6.25 mM SCFAs, additional organic acids are required to achieve a pH of 4.7 after the sugars start fermenting during the long incubation time required for INAC.

Nonionized acids are considered to present high pKa values at pH 4.7, which enables them to induce INAC. The mixture of 5 mM butyric acid, 15 mM propionic acid and 40 mM acetic acid induced similar INAC areas in *A. oris* MG1 and the *A. oris* MG1 Δ*fimA* mutant to those induced by 60 mM butyric acid. According to the Stephan curve, the pH in dental plaques decreases to less than 5.0 after every meal [33], and at least a 60 mM concentration of SCFAs is required to decrease the pH to that level. Therefore, 60 mM (pH 4.7) is a very important threshold because it presents clinical relevance for caries development and the enhancement of *A. naeslundii* and *A. oris* colonization*.* Benevolent organisms such as *V. parvula*, which is an early colonizer on the tooth surface, might support the INAC of *A. oris* because it catabolizes lactate produced by streptococci into SCFAs with high pKa values, such as propionic and acetic acids, with a reduced ability to solubilize enamel [30]. Such metabolic cooperation among bacteria is crucial for the establishment of oral biofilms [40]. Taken together, these results indicate that nonionized acids in the mixture of SCFAs (such as those with high pKa values) exuded from the biofilm as the pH reaches 4.7 will disperse and reinforce biofilm formation by enhancing the new attachment and aggregation of *A. naeslundii* and *A. oris*. Moreover, organic acids in the plaque might induce the readhesion and colonization of *A. naeslundii* and *A. oris* in the subgingival area and periodontal pocket without streptococci. This scenario is supported by a report that *Actinomyces* spp. are a prominent component of the periodontal flora [41].

A previous report showed that *Bacillus subtilis* death results from a change in the intracellular pH caused by the passage of weak organic acids, such as acetic acid, across the cell membrane when incubated with *B. subtilis* at a pH value near the pKa (4.7) [42]. Weak acids can permeate bacterial membranes more readily than strong acids, such as HCl and lactic acid, because of the equilibrium between their ionized and nonionized forms at the pKa [43]. These nonionized forms pass through the bacterial membrane and induce stress in cells. In contrast, lactic acid cannot undergo conversion to its nonionized form from its ionized form at a pH of 4.7, and although the ionized form affects fimbrillins on the bacterial membrane surface, strong acids cannot pass through the membrane into the intracellular space. The type 1 and type 2 fimbriae-dependent INAC of *A. oris* MG1 live cells was greatly enhanced by treatment with each SCFA at 60 mM or with a mixture of the SCFAs with high-pKa organic acids (acetic, butyric and propionic acids) at a total concentration of 60 mM. Conditions in which the pH was brought to 4.7 with HCl, in which only the ionized acid form is generated, induced greater INAC of *A. oris* MG1 live cells together with dead cells than was induced by 60 mM butyric acid. The ionized form present at low pH causes cytoplasmic membrane damage to a major barrier to proton influx [44]. Proteins embedded in cell membranes are exposed to the detrimental effect of the acidic pH at their cellular location and inactivated [45, 46]. Fimbrillin-dependent and fimbrillin-independent INAC in live cells is considered to rely on the effects of nonionized organic acids on the membrane surface of the fimbriae in dental plaques. In contrast, the ionized form induces cell death through membrane damage, resulting in the dead cell-dependent initial adherence and aggregation of *A. oris* in lower-pH conditions.

As we previously reported, this SCFA-induced increase in biofilm formation by *A. naeslundii* is dependent on the expression of GroEL, a stress response protein [36]. The INAC of *A. naeslundii* induced by 60 mM butyric acid is also GroEL-dependent [37]. The INAC of *A. oris* MG1 Δ*fimA* cells stimulated with 60 mM butyric acid was inhibited by the anti-GroEL antibody. Therefore, the nonionized acid form of acids with high pKa values may be critical for GroEL-dependent and FimA-independent INAC. According to these results, the mechanism of the INAC of *A. naeslundii* × 600 might be similar to that of the *A. oris* MG1 Δ*fimA* mutant treated with 60 mM butyric acid and may not be dependent on fimbriae*.*

GroEL is a member of a group of heat shock proteins that includes HrcA, GrpE and DnaK, which are stress-responsive molecular chaperones in bacteria [43, 47, 48]. These proteins control protein misfolding and aggregation and promote protein refolding and proper assembly during conditions of stress [49]. Chaperones and stress response proteins may also act as microbial virulence factors when expressed at the cell surface, functioning as bacterial adhesins and promoting host tissue damage [49]. GroEL is exported outside of cells in membrane vesicles (MVs) [50]. Moreover, GroEL has been identified in MVs isolated from the culture medium of *Propionibacterium acnes* and *Acinetobacter baumannii* [51, 52]. In some probiotic lactobacilli, GAPDH, GroEL and DnaK have been identified as surface-associated moonlighting proteins that promote adherence and biofilm formation [53–55]. Under stress conditions, including the presence of butyric acid and propionic acid, MVs containing GroEL may be produced and connect live *A. oris* cells to solid surfaces.

*S. gordonii* and *S. sanguinis*, which are oral streptococci that commonly occur as early colonizers on the tooth surface, communicate with other oral bacteria and contribute to biofilm formation [56, 57]. *S. gordonii* and *S. sanguinis* express various antigenic types of cell wall polysaccharides on their cell surfaces. These polysaccharides serve as receptors for the Gal/GalNAc-reactive fimbrial lectin of *A. naeslundii* [58, 59]. Such interactions between the fimbriae of *Actinomyces* spp. and the polysaccharides of oral streptococci lead to the initiation of biofilm formation as well as biofilm development in multiple species in vivo and in vitro. However, SCFAs did not affect the interaction between the FimA of *A. oris* and *S. sanguinis* and principally induced the INAC of *A. oris*. Therefore, SCFAs and metabolites from other oral bacteria do not enhance the communication between *Actinomyces* spp. and oral streptococci such as *S. sanguinis*, *S. gordonii*, and *S. mitis*, which are associated with the development of biofilms.

In conclusion, among SCFAs, butyric acid, propionic acid, acetic acid and a mixture thereof stimulate the type 1- and type 2 fimbrillin-dependent INAC of *A. oris* live cells. The effects are dependent on the levels of the nonionized forms of weak organic acids. The INAC associated with the interactions of *A. oris* with oral streptococci [24] is FimA-dependent but is not mediated by SCFAs. The ionized acid form of SCFAs is properly utilized by fimbrillins and dead cells to induce INAC, and the nonionized acid forms that cross the cell membrane cause cell stress and increase the INAC of live cells. GroEL was also found to be a contributor to the FimA-independent INAC of live *A. oris* cells stimulated by nonionized acid. These results provide new mechanisms of fimbrillin-dependent and fimbrillin-independent INAC without physical interaction with oral streptococci in the communication between *A. oris* and the oral biofilm bacteria that produce SCFAs. However, our study does not fully elucidate the causal molecules involved, and further study of the molecular functions of fimbrillins, ionized and nonionized SCFA forms, and GroEL expression is necessary to understand the INAC of *A. oris*. Clinical studies using materials to trap these organic acids are also required to advance oral hygiene to the next level.

## Conclusions

Among the SCFAs, weak organic acids: butyric acid, propionic acid, and acetic acid, which are produced in dental plaque, stimulate the type 1- and type 2 fimbrillin-dependent INAC of *A. oris* live cells. The effects are dependent on the levels of the non-ionized acid forms. The INAC associated with the interactions of *A. oris* with *S. sanguinis* or *S. gordonii* is FimA-dependent but is not mediated by the SCFAs. GroEL was also found to be a contributor to the FimA-independent INAC by live *A. oris* cells stimulated with non-ionized acid. These results provide new mechanisms for fimbrillin-dependent and fimbrillin -independent INAC in the communication of *A. oris* and the oral biofilm bacteria that produce SCFAs.

## Methods

### Human saliva collection

Un-stimulated human saliva samples were collected from 3 healthy volunteers (22 ~ 27 years old) and pooled into sterile bottle over a period of 5 min. The samples were centrifuged at 10,000×g for 10 min at 4 °C. After centrifugation, the supernatants were transferred into new sterile tubes and sterilized by a 0.22-μm Millex-GP filter (Merck Millipore, Bedford, MA, USA). The sterilized human saliva samples were stored at − 20 °C until use.

### INAC assays with monocultures or cocultures

The INAC of monocultures of the *A. oris* MG1 strain, MG1 fimbriae deletion mutants (Δ*fimP,* Δ*fimQ,* Δ*fimA* and Δ*fimB*) (Table 1) and streptococci (*Streptococcus sanguinis* ATCC10556*, Streptococcus gordonii* ATCC10558 and *Streptococcus salivarius* HT9R) and that of cocultures of *A. oris* MG1 or MG1 fimbriae deletion mutants with streptococci was assayed using the methods described in Arai et al. [37] with some modifications. First, the bacterial cultures of *A. oris* MG1 or MG1 fimbriae deletion mutants were washed with sterile PBS. Before the inoculation of the bacteria, 6-well culture plates (IWAKI Microplate; AGC, Tokyo, Japan) were coated with whole human saliva at 4 °C overnight and washed with sterile PBS. Each strain of bacteria was diluted with fresh Tryptic Soy Broth (TSB), and the concentrations of the bacterial cells were then adjusted to an optical density of 0.4 at 600 nm using fresh TSB with 0.25% sucrose. Then, 350 μl of the *A. oris* MG1 or fimbriae deletion mutant dilutions was mixed into 2,650 μl of TSB with 0.25% sucrose, and this mixture was applied to the human saliva-coated six-well culture plate. After preincubation at 37 °C for 3 h in an aerobic atmosphere, the bacterial cultures were removed, and the bottom of each well was gently washed with sterile PBS to remove the unattached cells. Incubation times greater than 3 h were not selected because the metabolic products present after incubation might affect colonization and biofilm formation. A concentration of at least 60 mM was required to achieve a low pH of 4.7 under the experimental conditions, and this concentration was selected as the test concentration of SCFAs because it is a key criterion for initial attachment and biofilm formation [37]. TSB with or without 60 mM SCFAs, 60 mM butyrate and 60 mM butyrate prepared with HCl at pH 4.7, 60 mM butyric acid prepared with NaOH at pH 7.2 or a mixture of 5 mM butyric acid, 15 mM propionic acid and 40 mM acetic acid was applied, and the cells were incubated for up to 1 h at 37 °C. After this incubation period, the bacterial cultures were removed and gently washed again with sterile PBS. The attached cells at the edge and bottom of the wells were stained with a FilmTracer LIVE/DEAD biofilm viability kit (Molecular Probes Inc., Eugene, OR) with final concentrations of 5 μM SYTO 9 and 30 μM propidium iodide. In a previous report [37], it was found that the bacteria were likely to physically adhere to and aggregate on the edge of the well after 3 h of incubation, and after removing planktonic cells by washing with PBS, the bacteria around the edge remained. The stained cells on the edge were observed with a confocal microscope {LSM 700 Meta NLO confocal laser scanning microscope (CLSM); Carl Zeiss Inc., Thornwood, NY}. The confocal images of INAC were visually observed using ZEN analysis software (Carl Zeiss). To calculate the percentages of the live and dead cell areas, triplicate assays were performed under each condition, and a typical image was selected from 3 images obtained with a confocal microscope. Three independent experiments were performed, and the 3 typical images of the initially attached cells from 3 independent experiments were analyzed using ImageJ 1.48 software.

**Table 1:** Bacterial strains used in this study

Strains	Characteristics	Reference
*A. oris* MG1	Type strain, possesses type 1 and 2 fimbriae	Wu *et al*., 2011 [23]
*A. oris* Δ*fimP*	Mutant of shaft fimbrillin of type 1	Mishra *et al*., 2010 [24]
*A. oris* Δ*fimQ*	Mutant of tip fimbrillin of type 1	Mishra *et al*., 2010 [24]
*A. oris* Δ*fimA*	Mutant of shaft fimbrillin of type 2	Wu *et al.*, 2011 [23]
*A. oris* Δ*fimB*	Mutant of tip fimbrillin of type 2	Wu *et al*., 2011 [23]

To determine the mechanism of the attachment and colonization of *A. oris* MG1 and MG1 Δ*fimA*, we added anti-GroEL polyclonal rabbit antibodies (diluted 1/4000; MBL: Medical & Biological Laboratories Co., Ltd., Nagoya, Japan) to the first preincubation medium for 3 h and to the second culture medium with SCFAs for 1 h [37]. An anti-glucan-binding protein C (GBPC) polyclonal rabbit antibody was used as a control [36]. The effects of the antibodies and FruA were assessed by staining the cells with the FilmTracer LIVE/DEAD biofilm viability kit and through visual observation using a CLSM.

### Counting of viable cell numbers in the initially attached and colonized cell cultures

Following the same cell preparation as used for the INAC assays, TSB with or without 60 mM SCFAs was applied, and the *A. oris* MG1 was incubated for as long as 4 h (pre-incubation for 3 h and incubation with SCFAs for 1 h) at 37 °C. After incubation, the bacterial cultures were removed, and the attached cells were gently rewashed with sterile PBS. The attached cells were then removed using a cell scraper (Sumitomo Bakelite Co. Ltd., Tokyo, Japan) and collected by pipetting them into a centrifugation tube. After washing with sterile PBS, the cells were diluted with sterile PBS and poured onto BHI agar plates. After 48 h of incubation in an aerobic atmosphere, the number of colonies was counted.

### Statistical analysis

The comparisons of biofilm areas and percentages of live and dead cell areas between two groups were performed using Student’s *t*-tests, with statistical significance defined as a *p*-value < 0.05. Data were analysed with Microsoft Excel and SPSS (IBM SPSS statistics 24; IBM corporation, Armonk, NY).

### Data availability statement

All relevant data are within the manuscript and its supporting information files.

## Supplementary information

**Additional file 1.**

## Data Availability

The datasets used and/or analysed during the current study are available from the corresponding author on reasonable request.

## Abbreviations

SCFAs: Short-chain fatty acids
INAC: Initial attachment and colonization
PRPs: Proline-rich proteins
CLSM: Confocal laser scanning microscope
GBPC: Glucan binding protein C
BHI: Brain heart infusion broth
