# Supplementary material for: Short chain fatty acids induced the type 1 and type 2 fimbrillin-dependent and fimbrillin-independent initial attachment and colonization of Actinomyces oris monoculture but not coculture with streptococci
Source: BMC Microbiol. 2020 Oct 31;20:329. doi: 10.1186/s12866-020-01976-4 (PMC7603776; doi:10.1186/s12866-020-01976-4)
Supplement: Supplementary file 1 — Additional file 1. [file 12866_2020_1976_MOESM1_ESM.pptx]

## Slide 1
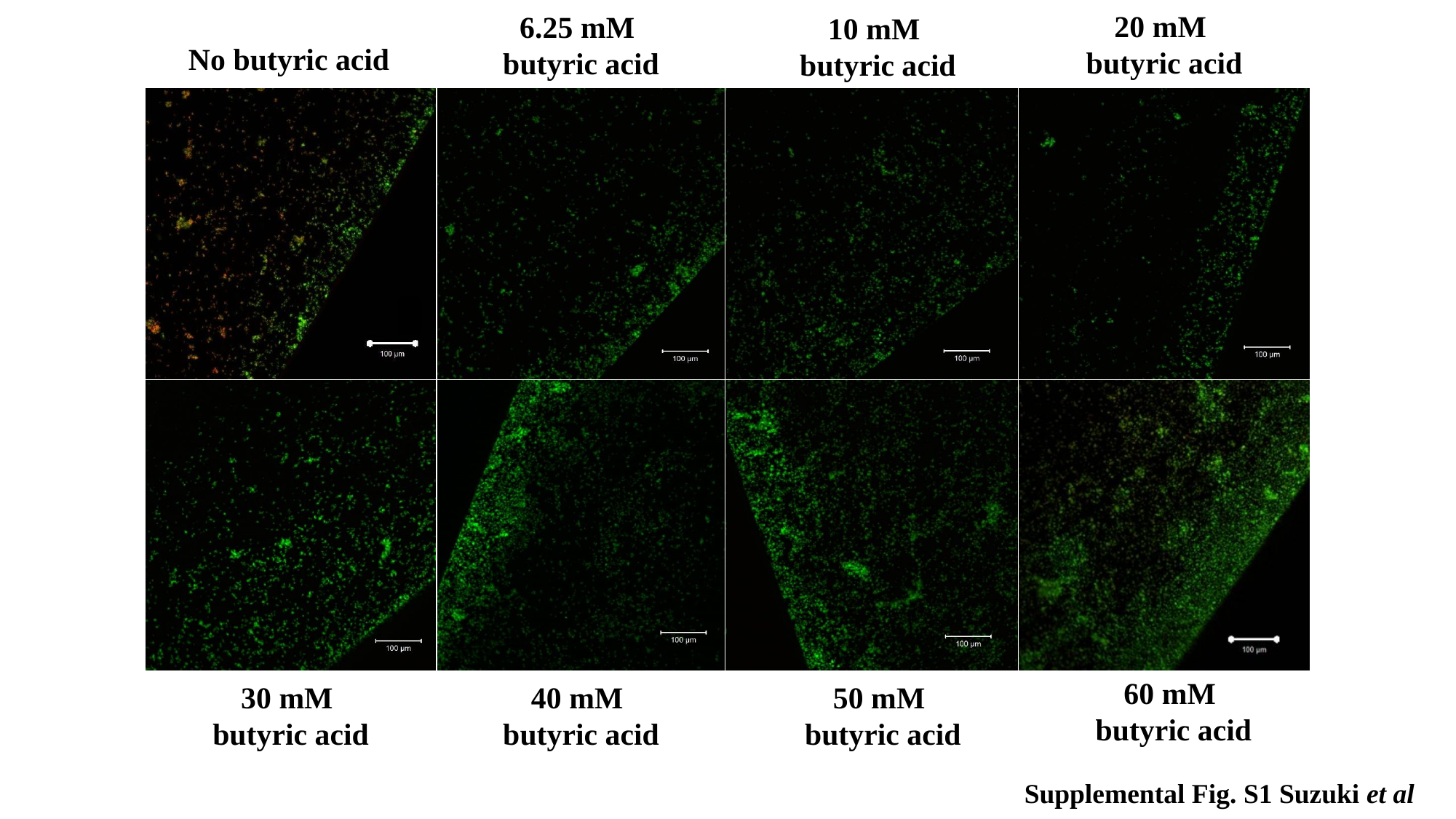

20 mM
butyric acid
6.25 mM
butyric acid
10 mM
butyric acid
No butyric acid
60 mM
butyric acid
30 mM
butyric acid
40 mM
butyric acid
50 mM
butyric acid
Supplemental Fig. S1 Suzuki et al

## Slide 2
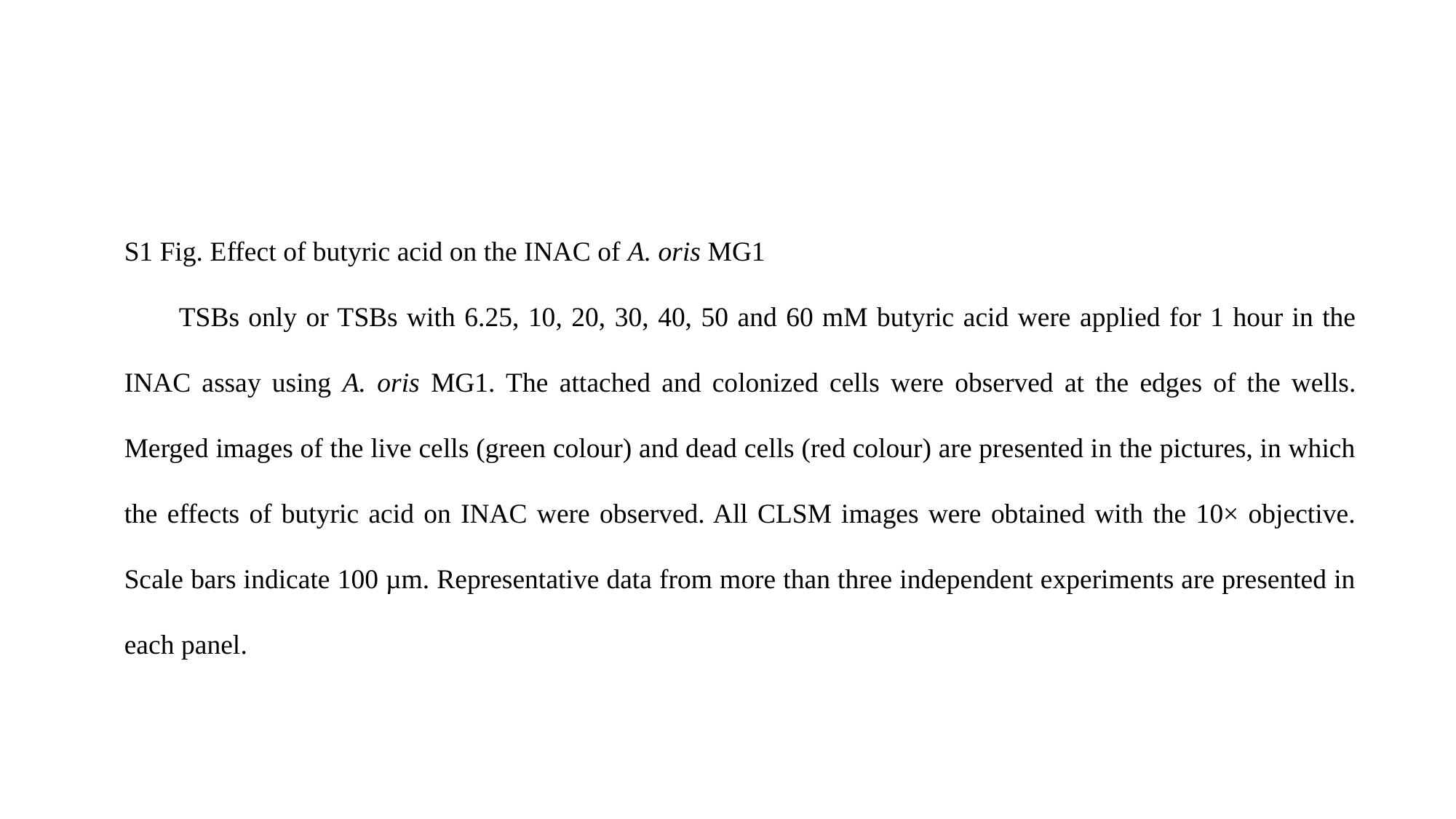

S1 Fig. Effect of butyric acid on the INAC of A. oris MG1
TSBs only or TSBs with 6.25, 10, 20, 30, 40, 50 and 60 mM butyric acid were applied for 1 hour in the INAC assay using A. oris MG1. The attached and colonized cells were observed at the edges of the wells. Merged images of the live cells (green colour) and dead cells (red colour) are presented in the pictures, in which the effects of butyric acid on INAC were observed. All CLSM images were obtained with the 10× objective. Scale bars indicate 100 µm. Representative data from more than three independent experiments are presented in each panel.

## Slide 3
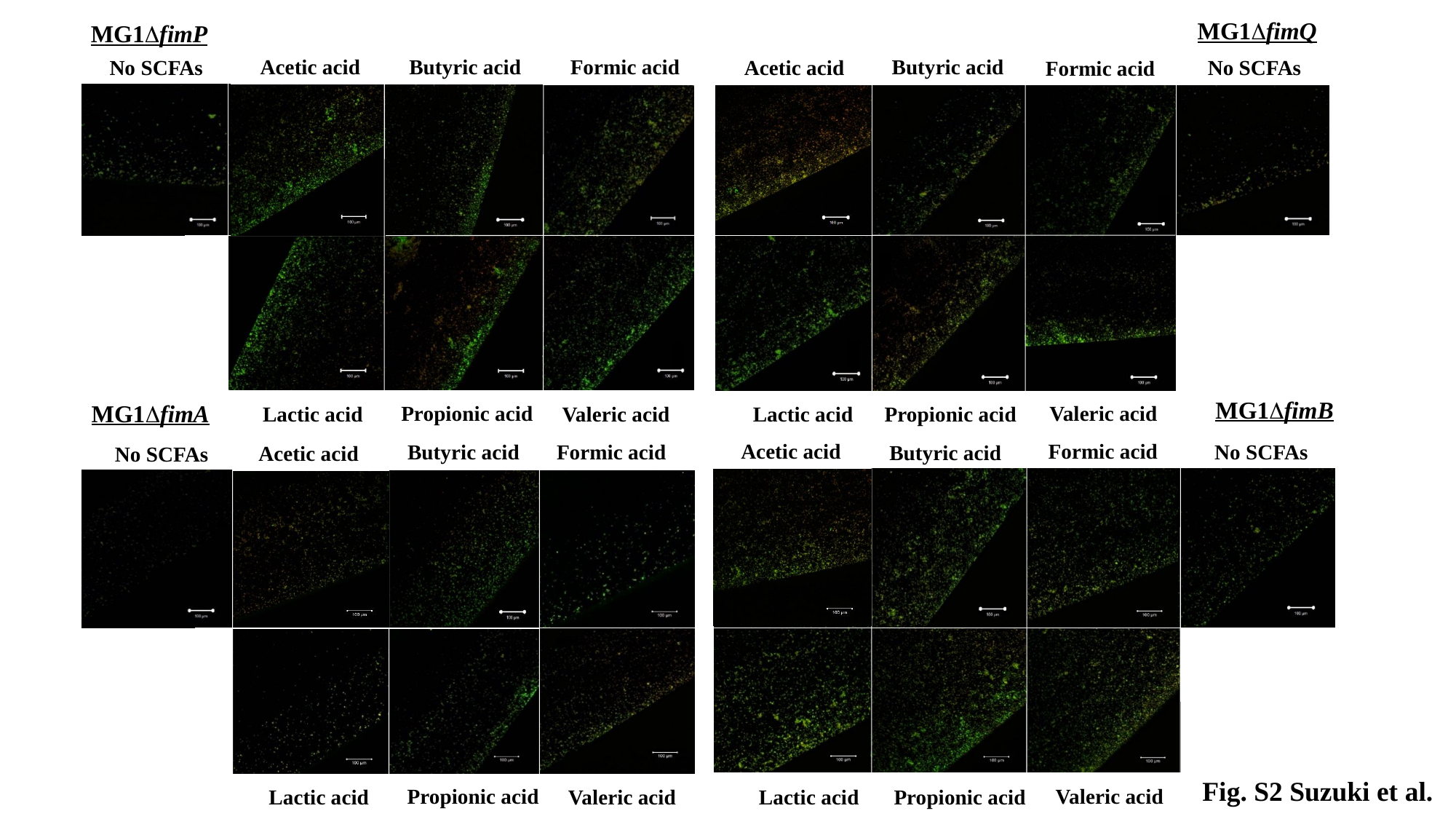

MG1DfimQ
MG1DfimP
Acetic acid
Butyric acid
Formic acid
Butyric acid
No SCFAs
No SCFAs
Acetic acid
Formic acid
MG1DfimB
MG1DfimA
Propionic acid
Valeric acid
Lactic acid
Lactic acid
Valeric acid
Propionic acid
Acetic acid
Formic acid
Formic acid
No SCFAs
Butyric acid
Butyric acid
Acetic acid
No SCFAs
G
Fig. S2 Suzuki et al.
Propionic acid
Valeric acid
Lactic acid
Lactic acid
Valeric acid
Propionic acid

## Slide 4
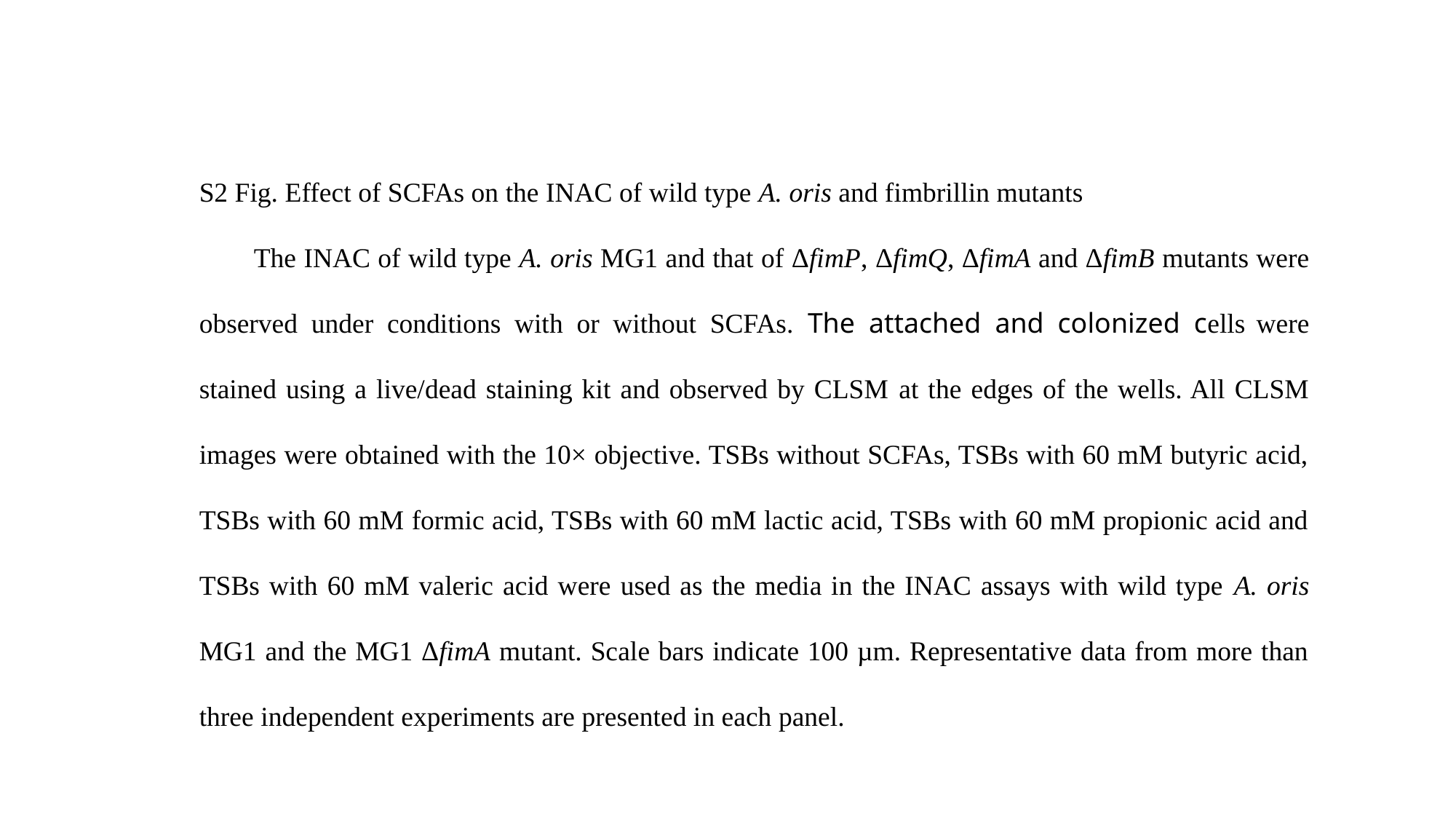

S2 Fig. Effect of SCFAs on the INAC of wild type A. oris and fimbrillin mutants
The INAC of wild type A. oris MG1 and that of ΔfimP, ΔfimQ, ΔfimA and ΔfimB mutants were observed under conditions with or without SCFAs. The attached and colonized cells were stained using a live/dead staining kit and observed by CLSM at the edges of the wells. All CLSM images were obtained with the 10× objective. TSBs without SCFAs, TSBs with 60 mM butyric acid, TSBs with 60 mM formic acid, TSBs with 60 mM lactic acid, TSBs with 60 mM propionic acid and TSBs with 60 mM valeric acid were used as the media in the INAC assays with wild type A. oris MG1 and the MG1 ΔfimA mutant. Scale bars indicate 100 µm. Representative data from more than three independent experiments are presented in each panel.

## Slide 5
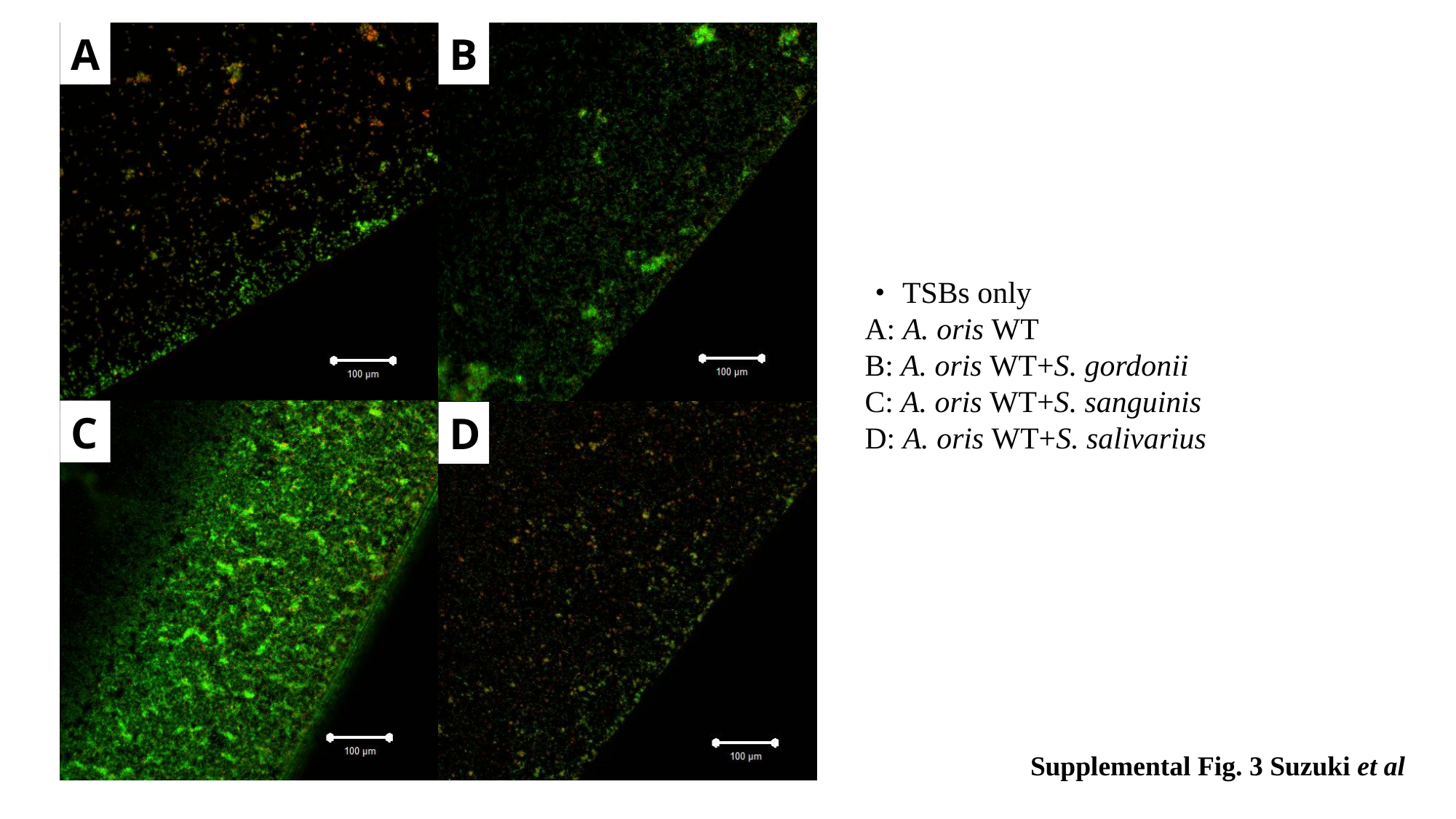

A
B
・TSBs only
A: A. oris WT
B: A. oris WT+S. gordonii
C: A. oris WT+S. sanguinis
D: A. oris WT+S. salivarius
C
D
Supplemental Fig. 3 Suzuki et al

## Slide 6
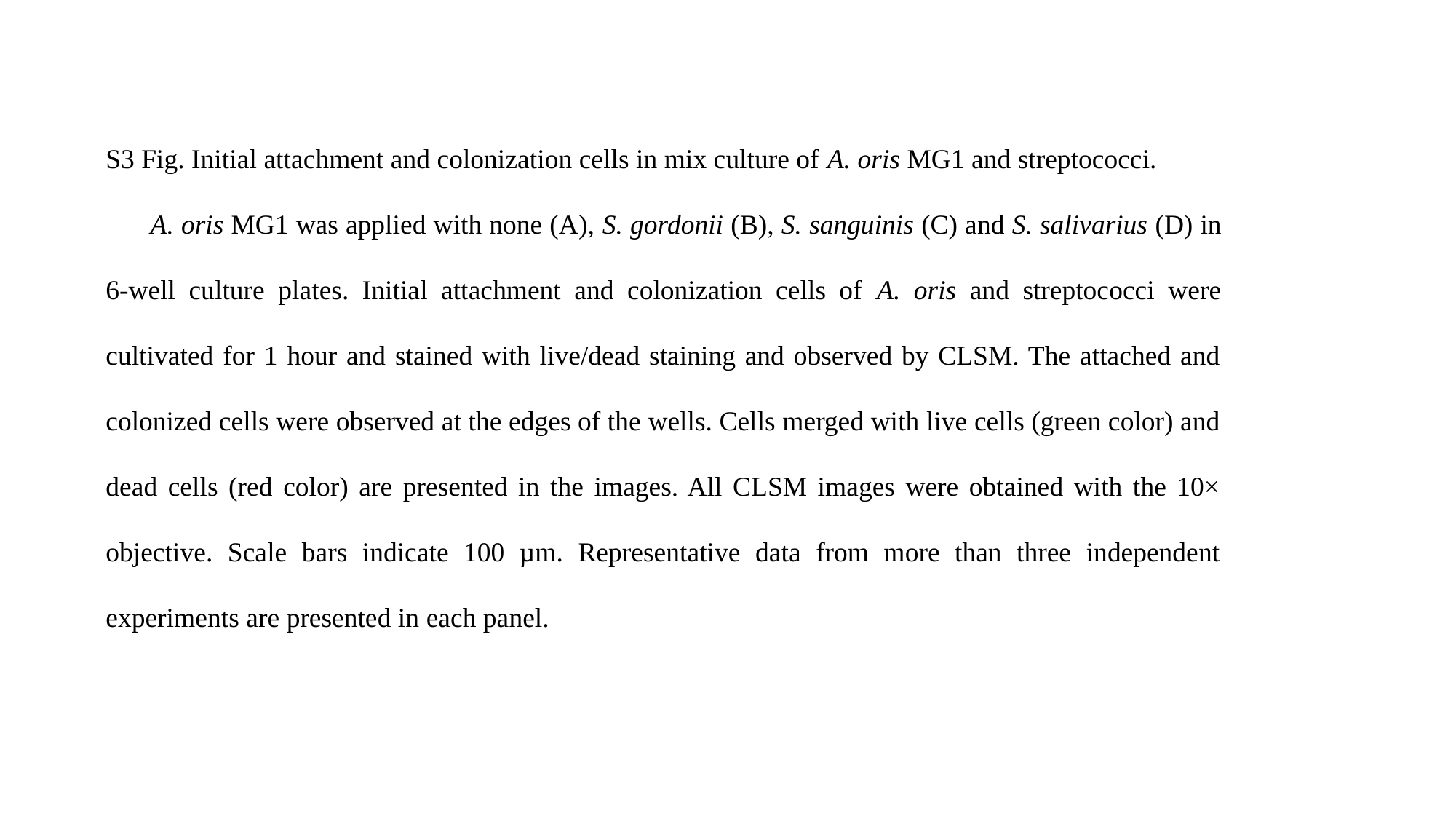

S3 Fig. Initial attachment and colonization cells in mix culture of A. oris MG1 and streptococci.
 A. oris MG1 was applied with none (A), S. gordonii (B), S. sanguinis (C) and S. salivarius (D) in 6-well culture plates. Initial attachment and colonization cells of A. oris and streptococci were cultivated for 1 hour and stained with live/dead staining and observed by CLSM. The attached and colonized cells were observed at the edges of the wells. Cells merged with live cells (green color) and dead cells (red color) are presented in the images. All CLSM images were obtained with the 10× objective. Scale bars indicate 100 µm. Representative data from more than three independent experiments are presented in each panel.

## Slide 7
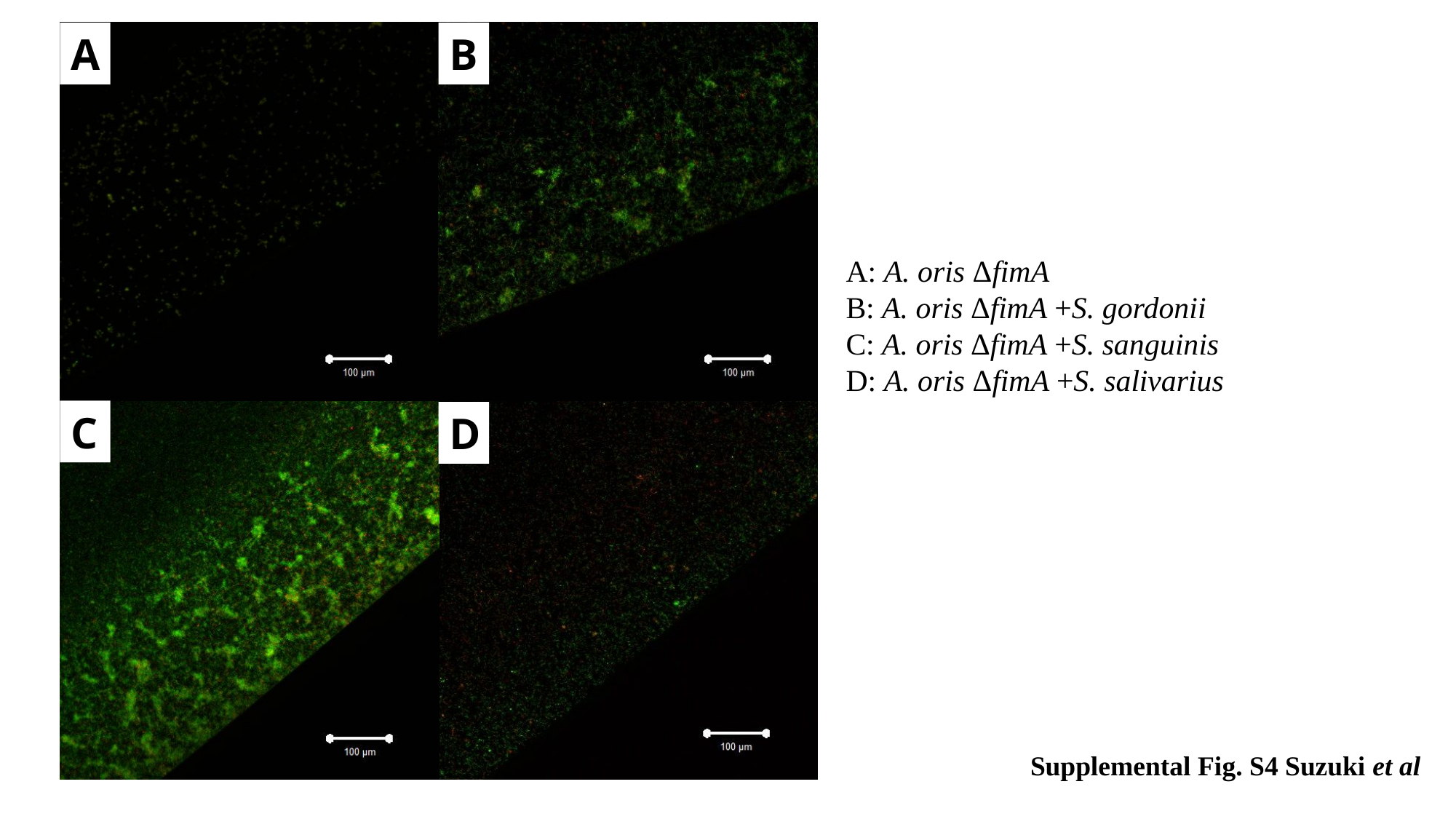

A
B
A: A. oris ΔfimA
B: A. oris ΔfimA +S. gordonii
C: A. oris ΔfimA +S. sanguinis
D: A. oris ΔfimA +S. salivarius
C
D
Supplemental Fig. S4 Suzuki et al

## Slide 8
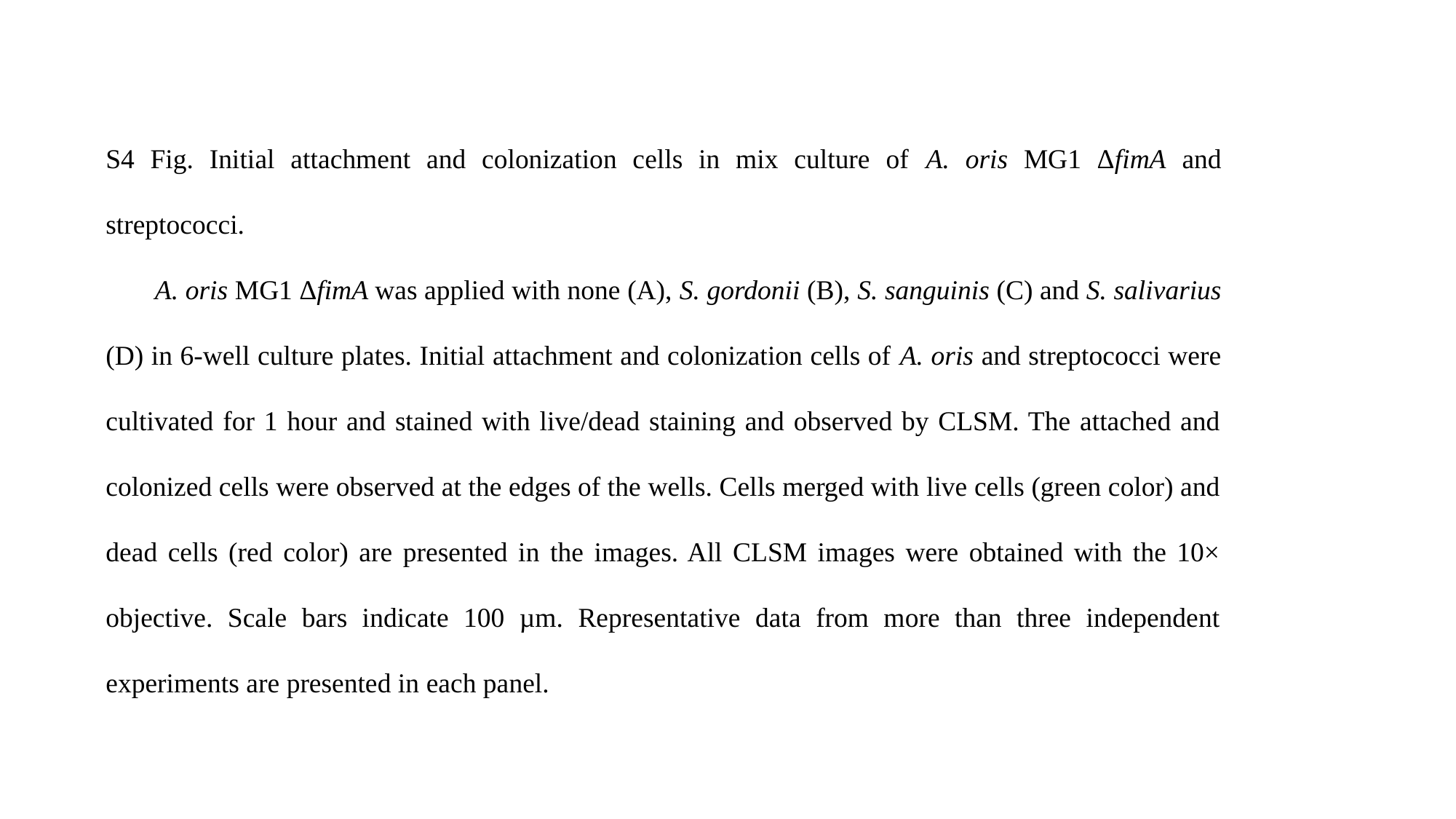

S4 Fig. Initial attachment and colonization cells in mix culture of A. oris MG1 ΔfimA and streptococci.
 A. oris MG1 ΔfimA was applied with none (A), S. gordonii (B), S. sanguinis (C) and S. salivarius (D) in 6-well culture plates. Initial attachment and colonization cells of A. oris and streptococci were cultivated for 1 hour and stained with live/dead staining and observed by CLSM. The attached and colonized cells were observed at the edges of the wells. Cells merged with live cells (green color) and dead cells (red color) are presented in the images. All CLSM images were obtained with the 10× objective. Scale bars indicate 100 µm. Representative data from more than three independent experiments are presented in each panel.

## Slide 9
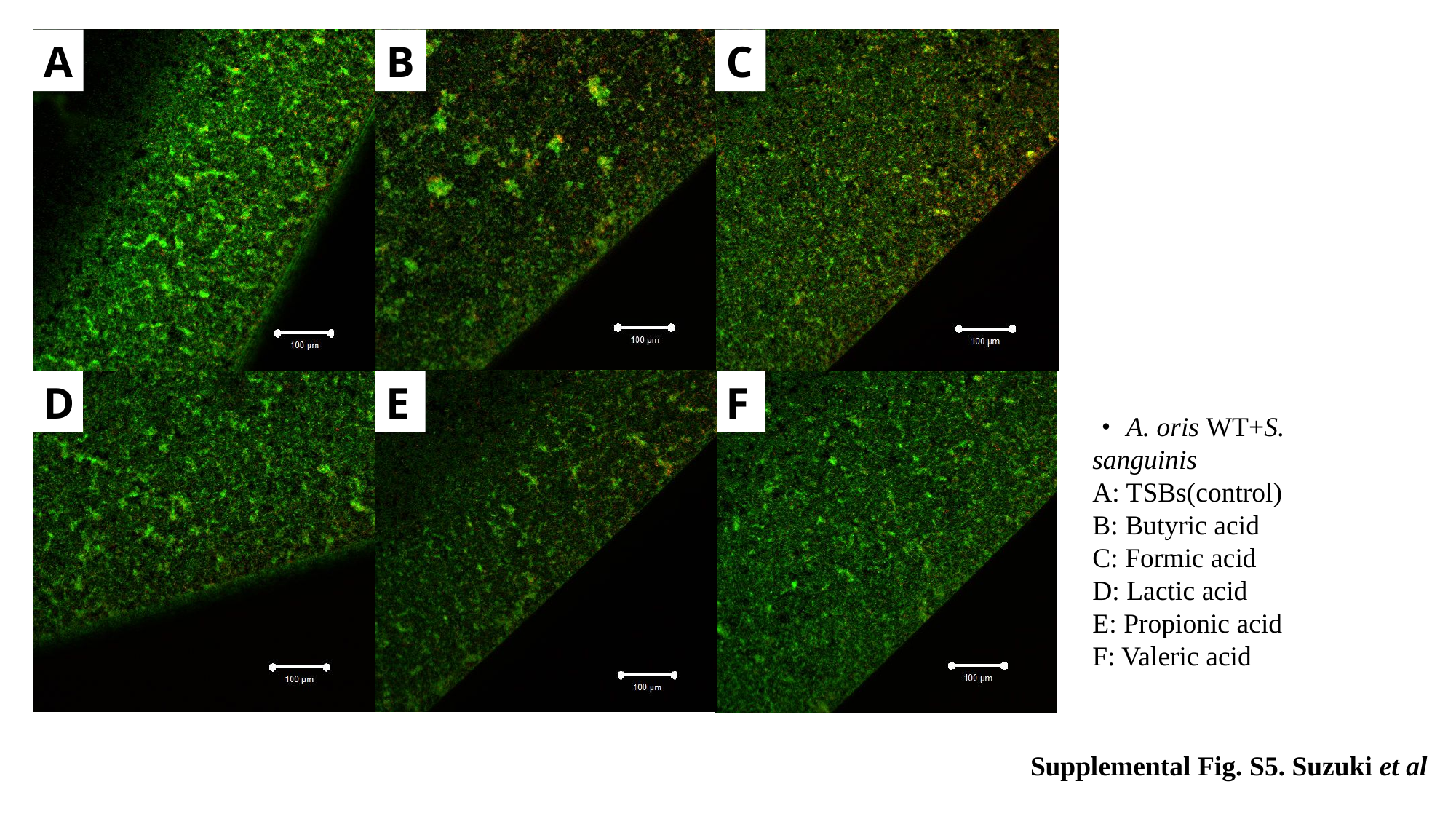

A
B
C
D
E
F
・A. oris WT+S. sanguinis
A: TSBs(control)
B: Butyric acid
C: Formic acid
D: Lactic acid
E: Propionic acid
F: Valeric acid
Supplemental Fig. S5. Suzuki et al

## Slide 10
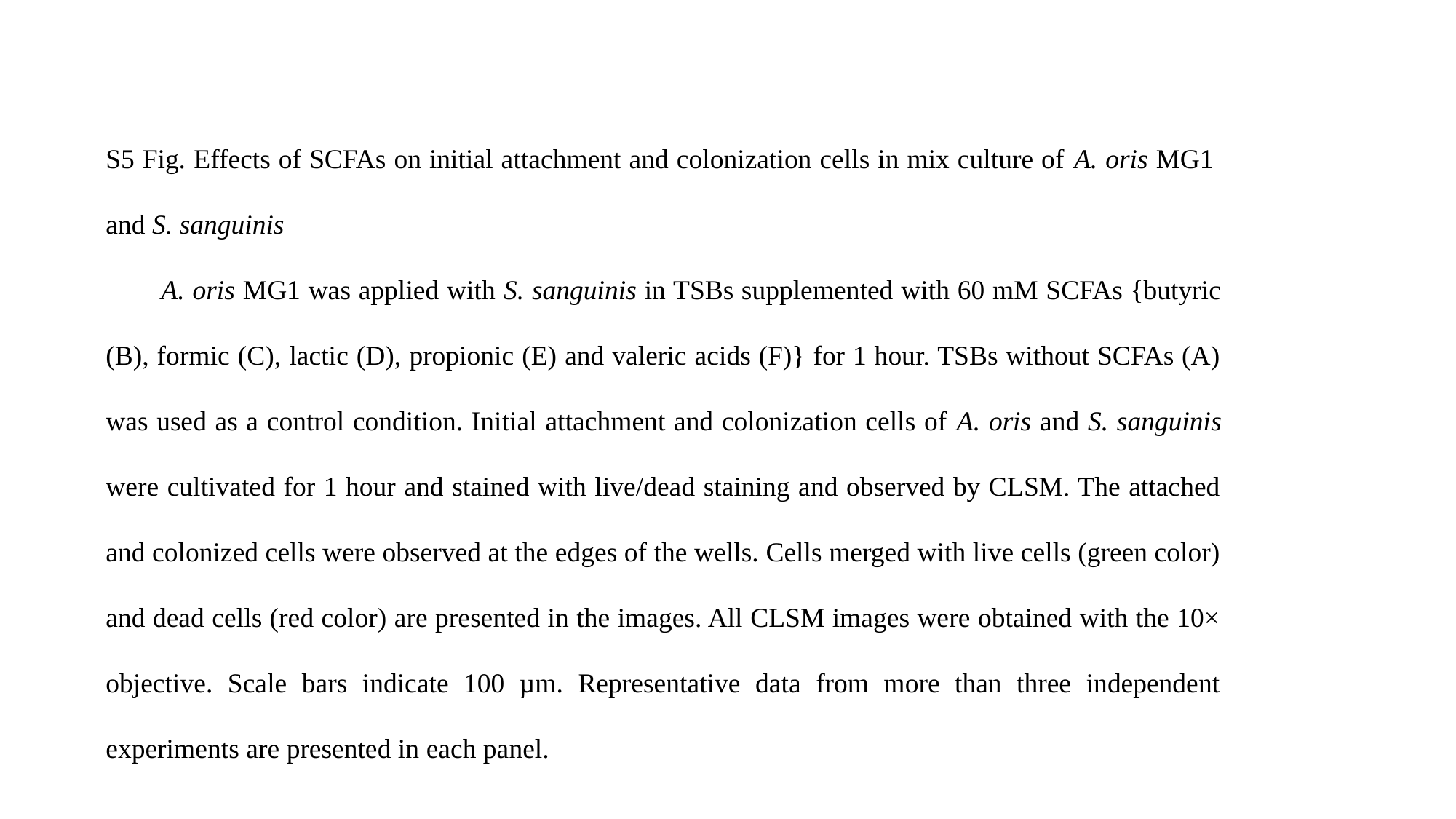

S5 Fig. Effects of SCFAs on initial attachment and colonization cells in mix culture of A. oris MG1 and S. sanguinis
 A. oris MG1 was applied with S. sanguinis in TSBs supplemented with 60 mM SCFAs {butyric (B), formic (C), lactic (D), propionic (E) and valeric acids (F)} for 1 hour. TSBs without SCFAs (A) was used as a control condition. Initial attachment and colonization cells of A. oris and S. sanguinis were cultivated for 1 hour and stained with live/dead staining and observed by CLSM. The attached and colonized cells were observed at the edges of the wells. Cells merged with live cells (green color) and dead cells (red color) are presented in the images. All CLSM images were obtained with the 10× objective. Scale bars indicate 100 µm. Representative data from more than three independent experiments are presented in each panel.

## Slide 11
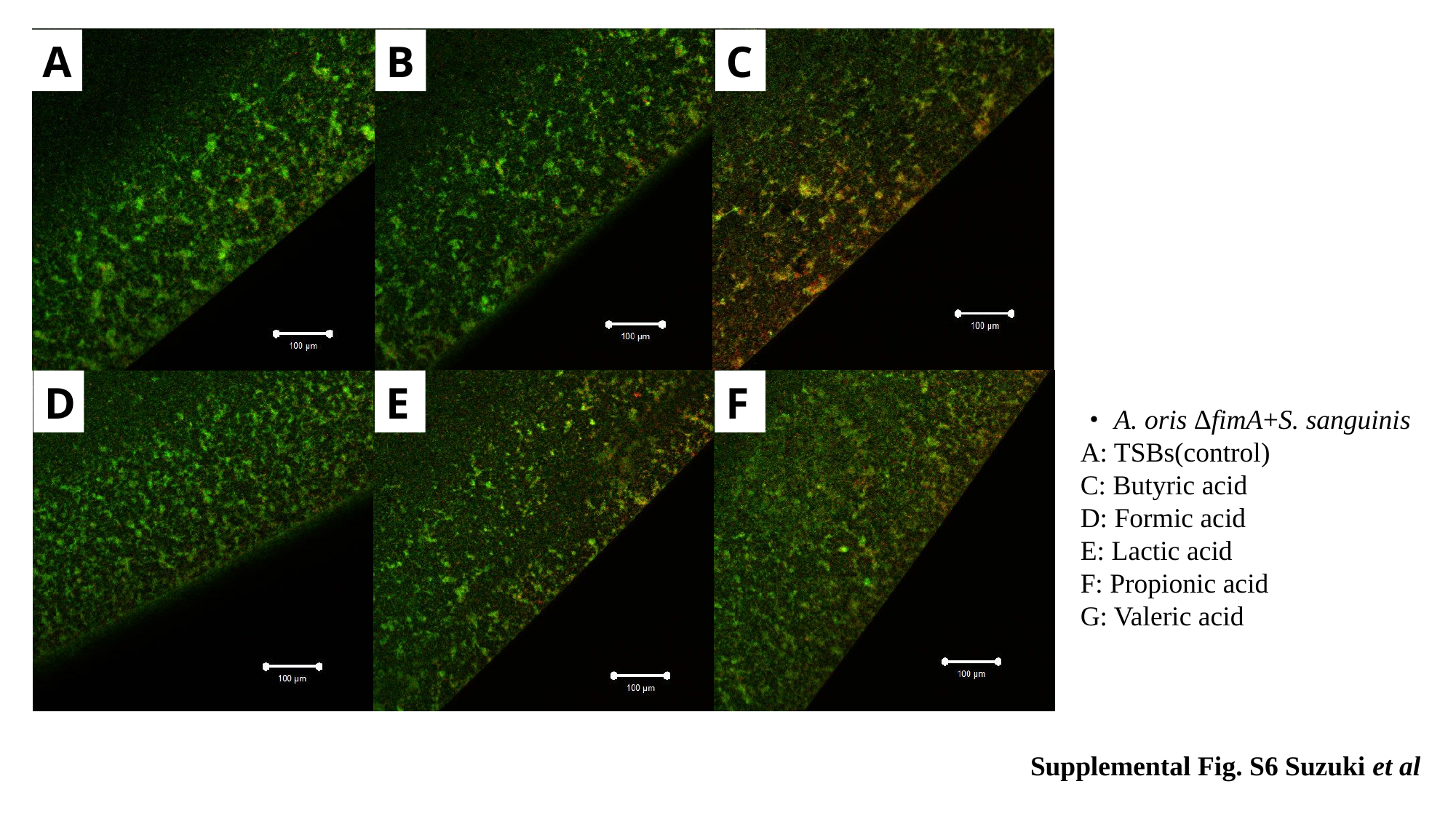

A
B
C
D
E
F
・A. oris ΔfimA+S. sanguinis
A: TSBs(control)
C: Butyric acid
D: Formic acid
E: Lactic acid
F: Propionic acid
G: Valeric acid
Supplemental Fig. S6 Suzuki et al

## Slide 12
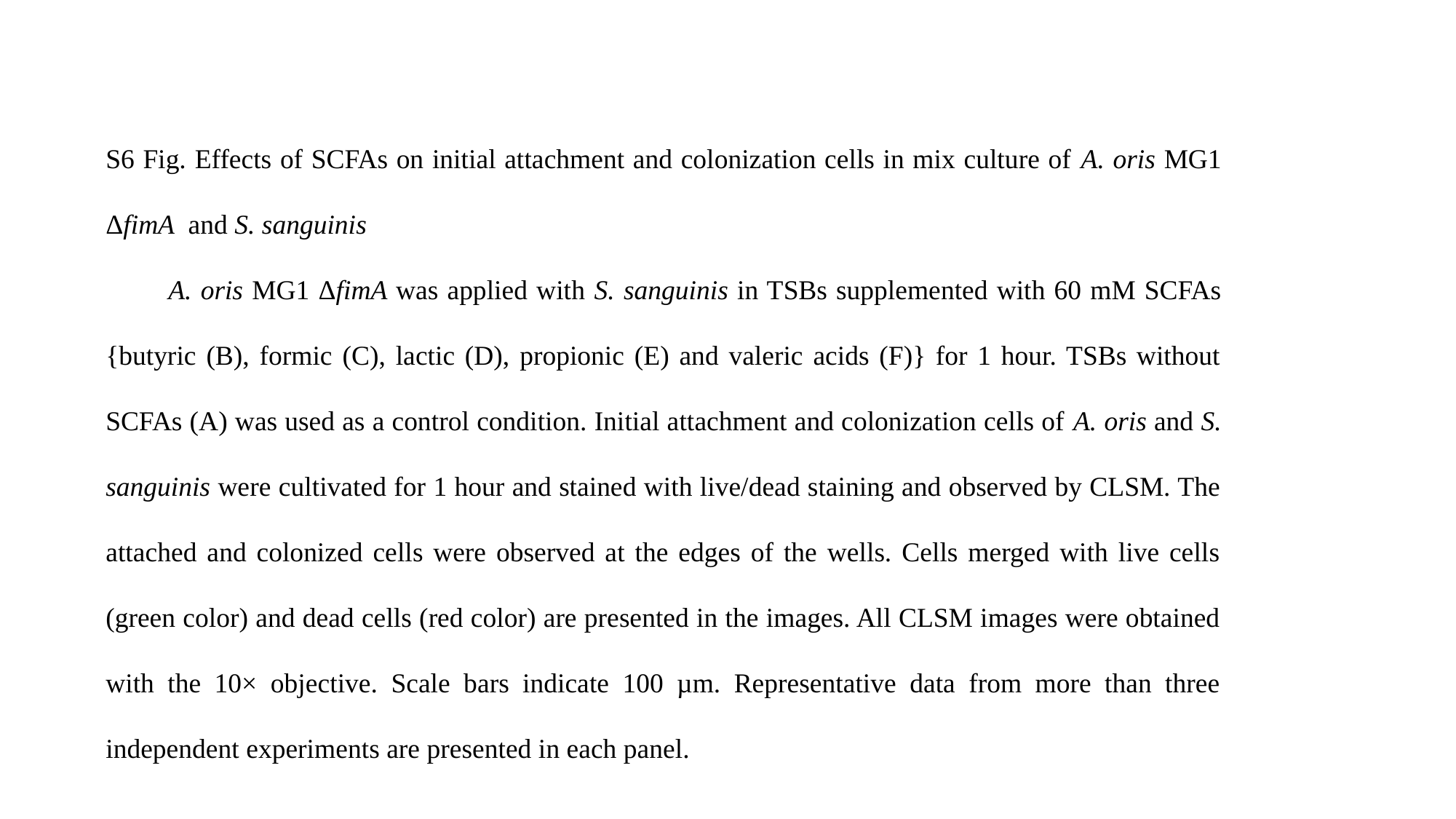

S6 Fig. Effects of SCFAs on initial attachment and colonization cells in mix culture of A. oris MG1 ΔfimA and S. sanguinis
 A. oris MG1 ΔfimA was applied with S. sanguinis in TSBs supplemented with 60 mM SCFAs {butyric (B), formic (C), lactic (D), propionic (E) and valeric acids (F)} for 1 hour. TSBs without SCFAs (A) was used as a control condition. Initial attachment and colonization cells of A. oris and S. sanguinis were cultivated for 1 hour and stained with live/dead staining and observed by CLSM. The attached and colonized cells were observed at the edges of the wells. Cells merged with live cells (green color) and dead cells (red color) are presented in the images. All CLSM images were obtained with the 10× objective. Scale bars indicate 100 µm. Representative data from more than three independent experiments are presented in each panel.

## Slide 13
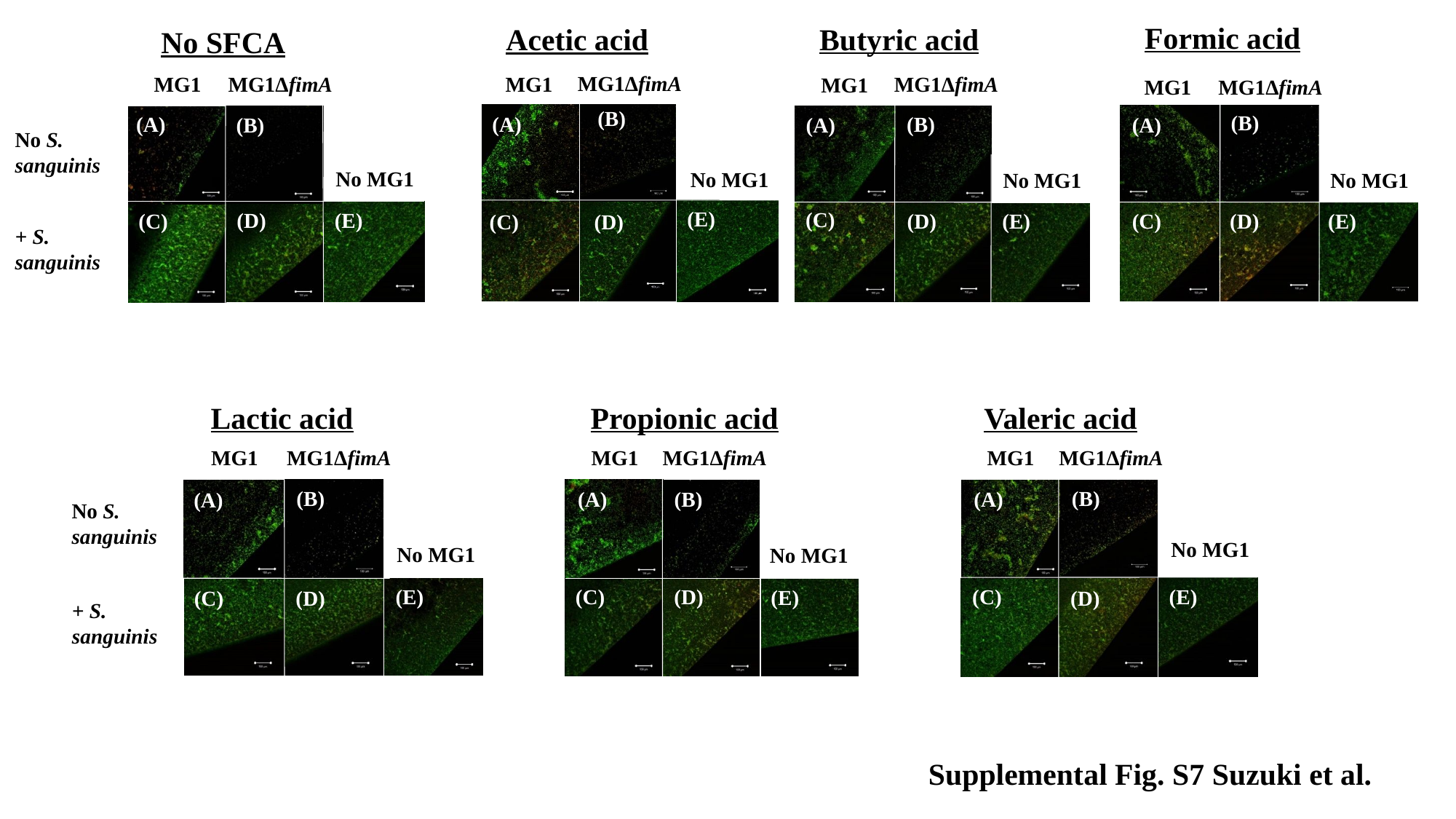

Formic acid
Butyric acid
Acetic acid
No SFCA
MG1ΔfimA
MG1ΔfimA
MG1
MG1
MG1ΔfimA
MG1
MG1
MG1ΔfimA
(B)
(B)
(A)
(B)
(A)
(A)
(A)
(B)
No S. sanguinis
No MG1
No MG1
No MG1
No MG1
(E)
(C)
(E)
(D)
(E)
(D)
(D)
(C)
(E)
(C)
(C)
(D)
+ S. sanguinis
Lactic acid
Propionic acid
Valeric acid
MG1ΔfimA
MG1
MG1ΔfimA
MG1
MG1
MG1ΔfimA
(B)
(B)
(A)
(B)
(A)
(A)
No S. sanguinis
No MG1
No MG1
No MG1
(C)
(E)
(D)
(C)
(E)
(E)
(C)
(D)
(D)
+ S. sanguinis
Supplemental Fig. S7 Suzuki et al.

## Slide 14
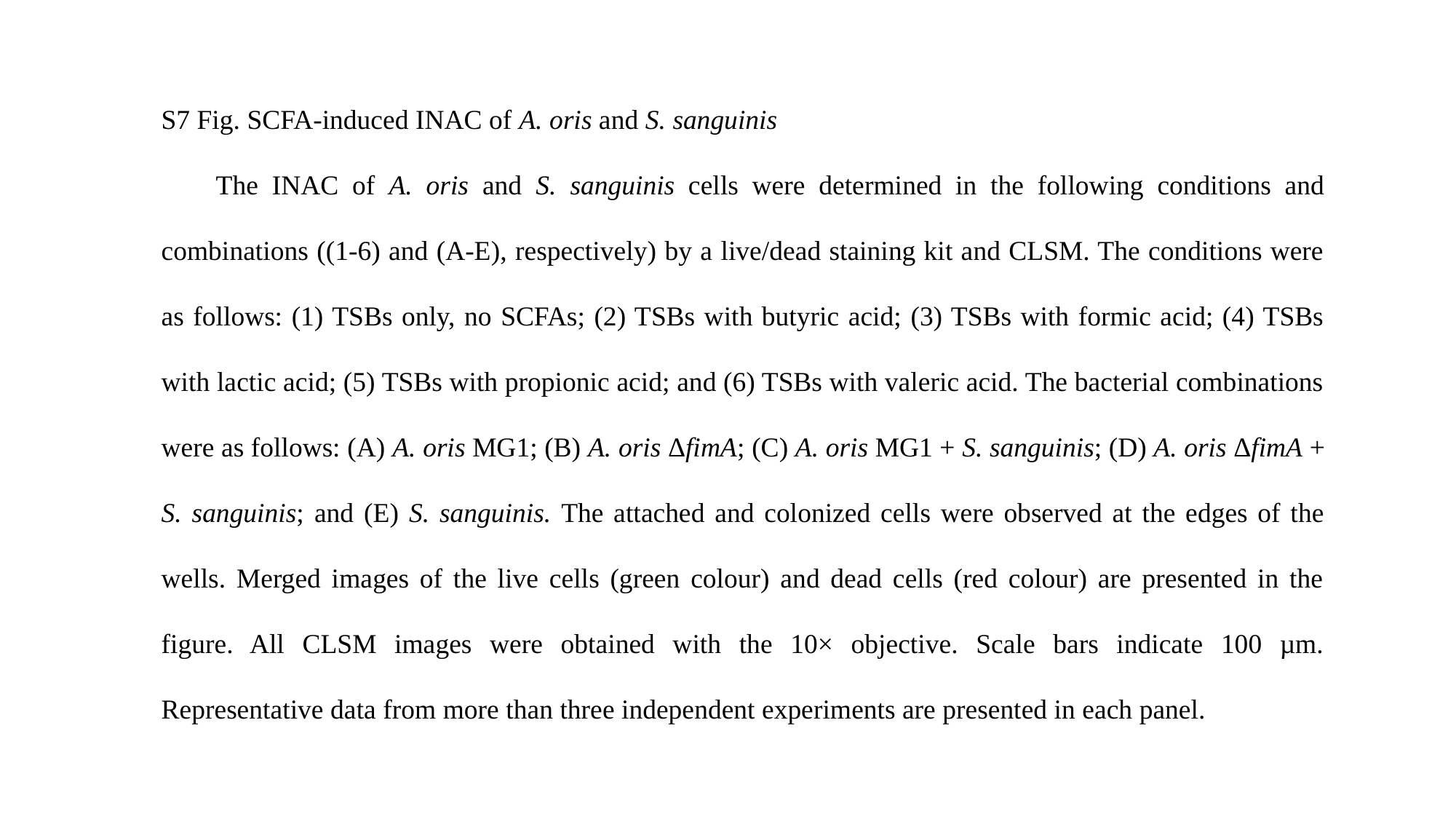

S7 Fig. SCFA-induced INAC of A. oris and S. sanguinis
The INAC of A. oris and S. sanguinis cells were determined in the following conditions and combinations ((1-6) and (A-E), respectively) by a live/dead staining kit and CLSM. The conditions were as follows: (1) TSBs only, no SCFAs; (2) TSBs with butyric acid; (3) TSBs with formic acid; (4) TSBs with lactic acid; (5) TSBs with propionic acid; and (6) TSBs with valeric acid. The bacterial combinations were as follows: (A) A. oris MG1; (B) A. oris ΔfimA; (C) A. oris MG1 + S. sanguinis; (D) A. oris ΔfimA + S. sanguinis; and (E) S. sanguinis. The attached and colonized cells were observed at the edges of the wells. Merged images of the live cells (green colour) and dead cells (red colour) are presented in the figure. All CLSM images were obtained with the 10× objective. Scale bars indicate 100 µm. Representative data from more than three independent experiments are presented in each panel.
